# Investigation of the Genome-Wide Genetic and Epigenetic Networks for Drug Discovery Based on Systems Biology Approaches in Colorectal Cancer

**DOI:** 10.3389/fgene.2020.00117

**Published:** 2020-03-06

**Authors:** Shan-Ju Yeh, Shuo-Wei Chen, Bor-Sen Chen

**Affiliations:** ^1^ Laboratory of Automatic Control, Signaling Processing and Systems Biology, Department of Electrical Engineering, National Tsing Hua University, Hsinchu, Taiwan; ^2^ Department of Pediatrics and Human Development, College of Human Medicine, Michigan State University, Grand Rapids, MI, United States

**Keywords:** colorectal cancer, systems biology, system identification, system modeling, system model selection, genome-wide genetic and epigenetic network, drug discovery

## Abstract

Colorectal cancer (CRC) is the third most commonly diagnosed type of cancer worldwide. The mechanisms leading to the progression of CRC are involved in both genetic and epigenetic regulations. In this study, we applied systems biology methods to identify potential biomarkers and conduct drug discovery in a computational approach. Using big database mining, we constructed a candidate protein-protein interaction network and a candidate gene regulatory network, combining them into a genome-wide genetic and epigenetic network (GWGEN). With the assistance of system identification and model selection approaches, we obtain real GWGENs for early-stage, mid-stage, and late-stage CRC. Subsequently, we extracted core GWGENs for each stage of CRC from their real GWGENs through a principal network projection method, and projected them to the Kyoto Encyclopedia of Genes and Genomes pathways for further analysis. Finally, we compared these core pathways resulting in different molecular mechanisms in each stage of CRC and identified carcinogenic biomarkers for the design of multiple-molecule drugs to prevent the progression of CRC. Based on the identified gene expression signatures, we suggested potential compounds combined with known CRC drugs to prevent the progression of CRC with querying Connectivity Map (CMap).

## Introduction

Colorectal cancer (CRC) is the third most commonly diagnosed type of cancer worldwide. Its incidence is increasing in young adults, especially in developing countries. As the fourth main cause of cancer-related deaths globally, CRC is a serious threat to human health (Deng et al., 2017). Recently, one study has mentioned that CRC was caused by epigenetic, genetic, and microenvironment factors (Khare and Verma, 2012). However, the molecular mechanisms of CRC are very complicated and remain unclear. Therefore, it is important to investigate the relationship between epigenetic and molecular mechanisms.

The progression mechanism and treatment methods for each stage of CRC has been discussed.

In the early stage of disease, CRC invades through the bowel wall, without involving the lymph nodes. The current treatment for early-stage CRC is surgical resection. Conducting surgical resection in this stage shows no evidence of subsequent influence on the lymph nodes of adjacent organs or distant sites (Freeman, 2013). In the mid stage, CRC involves the lymph nodes. At this stage, the main treatment method is surgical resection, followed by administration of chemotherapy. Using this strategy, a previous study reported that the overall survival of patients was improved and the CRC recurrence rate was decreased (Bos et al., 2015). In the late stage, CRC is widespread through metastases. At this stage, the main treatment methods focus on palliative chemotherapy and radiation therapy. Moreover, the survival rate in patients with late-stage CRC is usually minimal. It is necessary to regularly track the status of patients for the prevention of deterioration (Bouvier et al., 2015). Effective biomarkers, which are correlated with multiple drugs for patients in each stage of CRC, provide an alternative approach to treatment.

It is established that microRNAs (miRNAs), influenced by aberrant epigenetic regulation, also mediate the regulation of gene expression. miRNAs are a broad class of noncoding RNAs (length: ~21 nucleotides), which play crucial roles in posttranscriptional gene regulation (Lee and Ambros, 2001). Accumulating evidence indicates that dysregulations of miRNAs are crucial factors in human development and involved in human diseases, including CRC (Stefani and Slack, 2008). Several studies have investigated (Stefani and Slack, 2008; Chen et al., 2011) the effects of miRNAs on CRC. Nevertheless, further investigations regarding the molecular mechanisms involved in the development of CRC using genome-wide genetic and epigenetic networks (GWGENs), in which miRNAs participate, are warranted. In addition, long noncoding RNA (lncRNA) is a newly identified noncoding RNA molecules that are considered to be important regulators of tumor initiation and development (Xu et al., 2014). Although lncRNA has no biological functions on transcription, accumulating studies have uncovered the emerging role of lncRNA in multiple cellular processes, such as cell differentiation, proliferation, migration, invasion, apoptosis, and so on (Sun et al., 2015; Forrest et al., 2018; Xu et al., 2018; Di et al., 2019). Moreover, epigenetic changes modify the activation of certain genes, but not the genetic code sequence of DNA. Along with the accumulation of epigenetic changes in colon epithelial cells, these cells transform into adenocarcinomas (Lao and Grady, 2011). The most important epigenetic change is aberrant DNA methylation. All CRC cells have aberrantly methylated genes. Aberrant DNA methylation may interact with the change in the tumor microenvironment (TME). TME is the cellular environment in which the tumor exists, including the surrounding blood vessels, immune cells, fibroblasts, bone marrow–derived inflammatory cells, lymphocytes, signaling molecules, and the extracellular matrix (Berraondo et al., 2012). Among them, immune cells are the vital cells which can defeat nascent tumors in the TME. Although tumor cells cannot be completely eradicated by the immune cells, the latter may control tumor growth (Wang et al., 2017). Hence, proper activation of the immune response is beneficial to patients in the early stage of disease.

In this study, we applied a big database mining method to construct candidate GWGENs. The real GWGENs, including a protein-protein interaction network (PPIN) (Hsu et al., 2008; Lin et al., 2010), gene regulatory network (GRN), transcriptional regulations by transcription factors (TFs), lncRNAs (Chen et al., 2011), miRNA inhibitions, and DNA methylations were constructed through system modeling and identification of mRNAs and miRNAs expression using microarray data for each stage of CRC. Analysis of the real GWGENs is complicated; thus, we propose a principal network projection (PNP) method for the selection of the corresponding core GWGENs. Through denotation using the Kyoto Encyclopedia of Genes and Genomes (KEGG) pathways, the core GWGENs at the three stages of CRC cells could be projected into core pathways of the corresponding CRC cells. By comparing the core pathways of neighboring stages, we identified essential biomarkers involved in the progression mechanisms of CRC cells. These biomarkers could be considered as drug targets for different stages of CRC. By querying Connectivity Map (CMap), multiple-molecule drugs were designed for the therapeutic treatment of CRC progression.

## Materials and Methods

### Overview of Constructing Candidate GWGEN and Real GWGENs for Identifying Essential Biomarkers in the Progression of CRC

**Figure 1:**
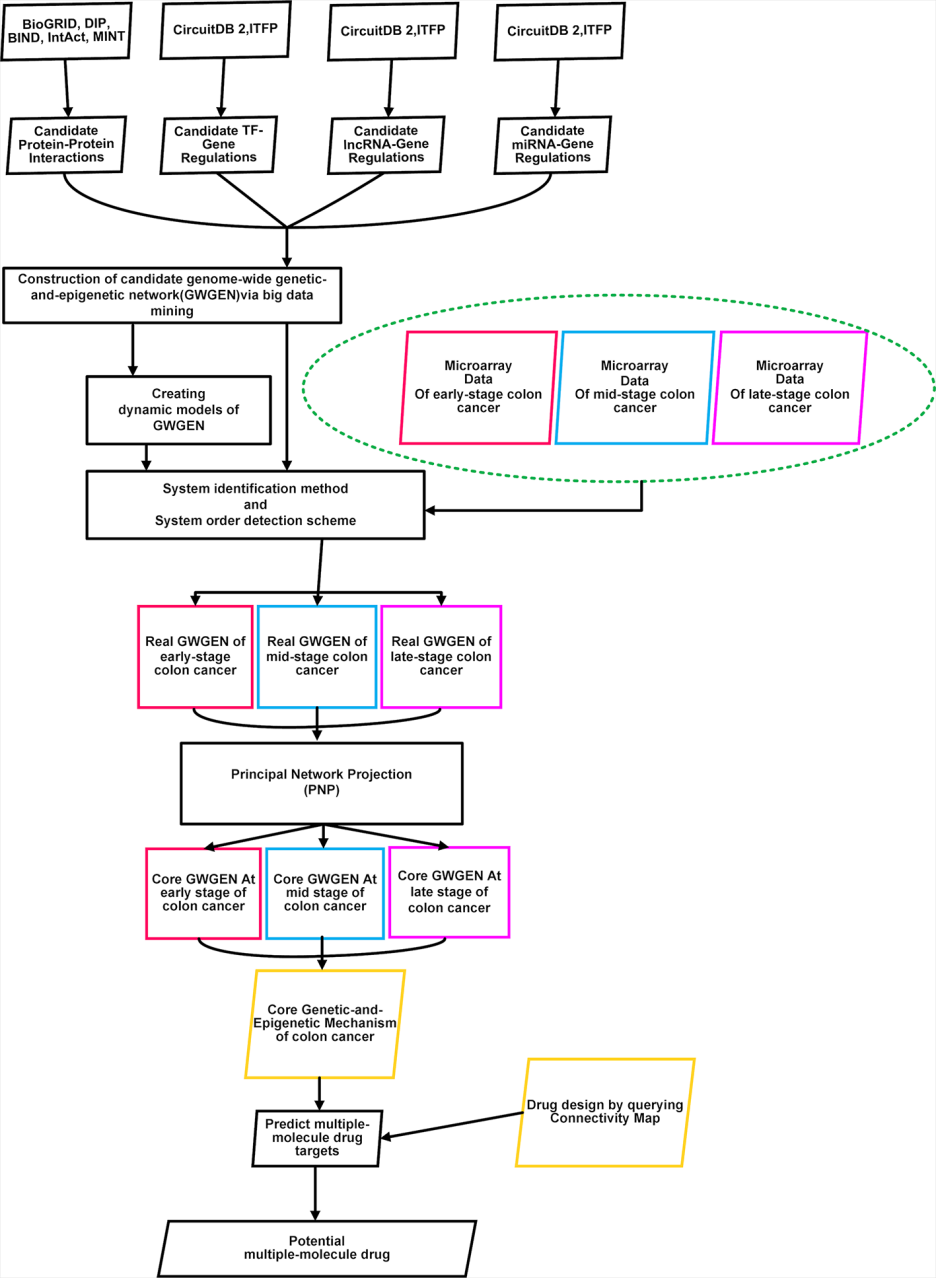
Flowchart of identifying essential biomarkers and finding potential multiple-molecule drug for three stages of colorectal cancer cells. Microarray data of early-stage to late-stage samples, miRNA data, miRNA database, TF-gene database, and BioGRID database were searched to construct candidate genome-wide genetic and epigenetic networks (GWGENs) consisting of a candidate gene regulatory network (GRN), candidate protein-protein interaction network (PPIN), and candidate miRNA regulation network. Subsequently, false positives of the candidate GWGENs were pruned to construct real GWGENs of early-stage, mid-stage, and late-stage colon cancer through system identification and system order detection methods. The core GWGENs of early-stage, mid-stage, and late-stage could be extracted from the corresponding real GWGENs using the principal network projection (PNP) method. We investigated different molecular mechanisms from early-stage to late-stage colon cancer according to their core pathways in the annotation of Kyoto Encyclopedia of Genes and Genomes (KEGG) pathways.

**Figure 2:**
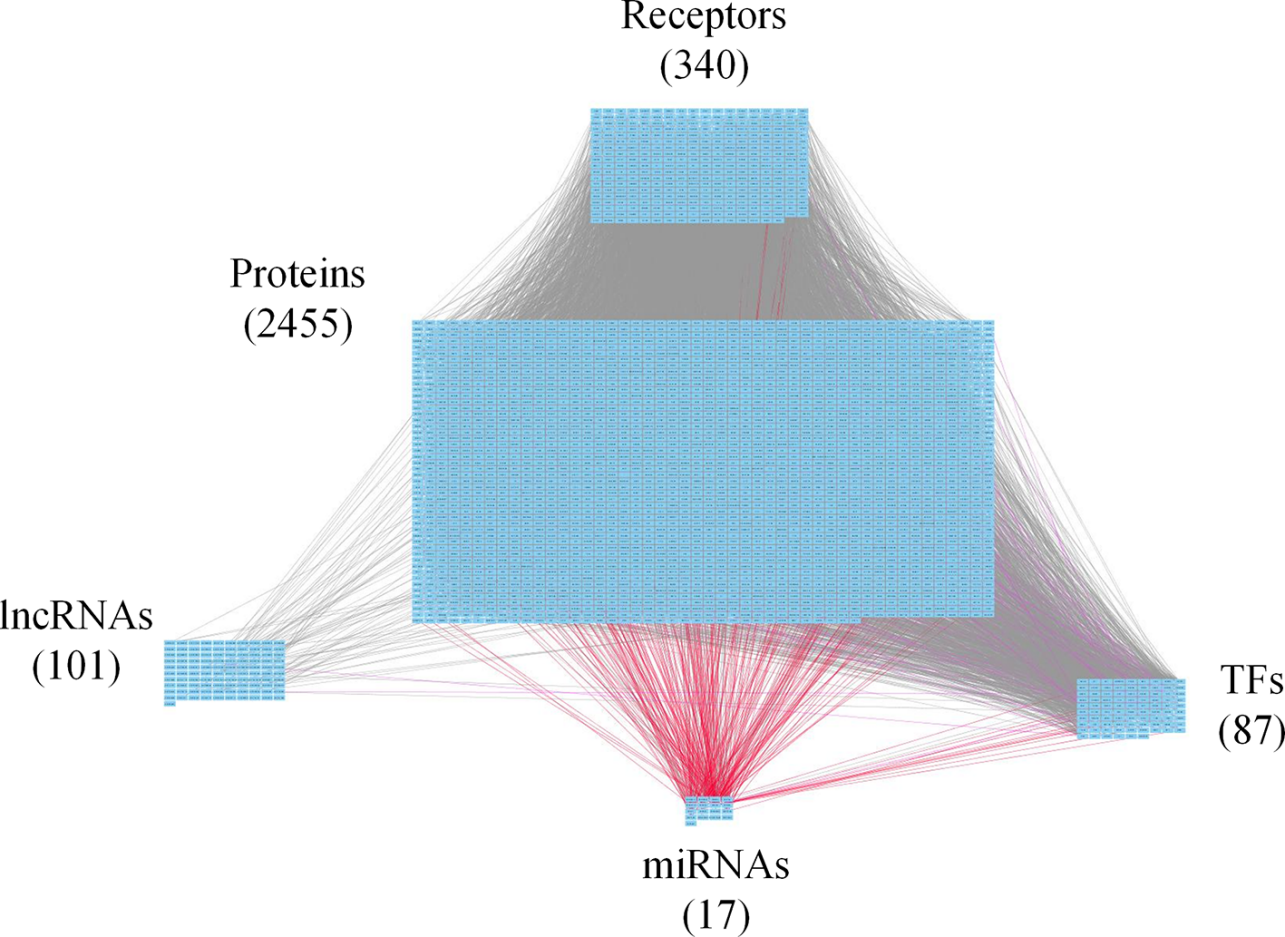
Core genome-wide genetic and epigenetic network (GWGEN) of early-stage colorectal cancer (CRC) cells. The core GWGEN of early-stage colon cancer was extracted from the corresponding real GWGEN by applying the principal network projection (PNP) method. Among the core GWGEN of early-stage CRC, there are 340 receptors, 2,455 proteins, 101 lncRNAs, 17 miRNAs, and 87 transcription factors (TFs). The gray lines represent edges in protein-protein interaction network (PPIN); the purple lines represent edges in gene regulatory network (GRN); the red lines represent miRNA regulations.

**Figure 3:**
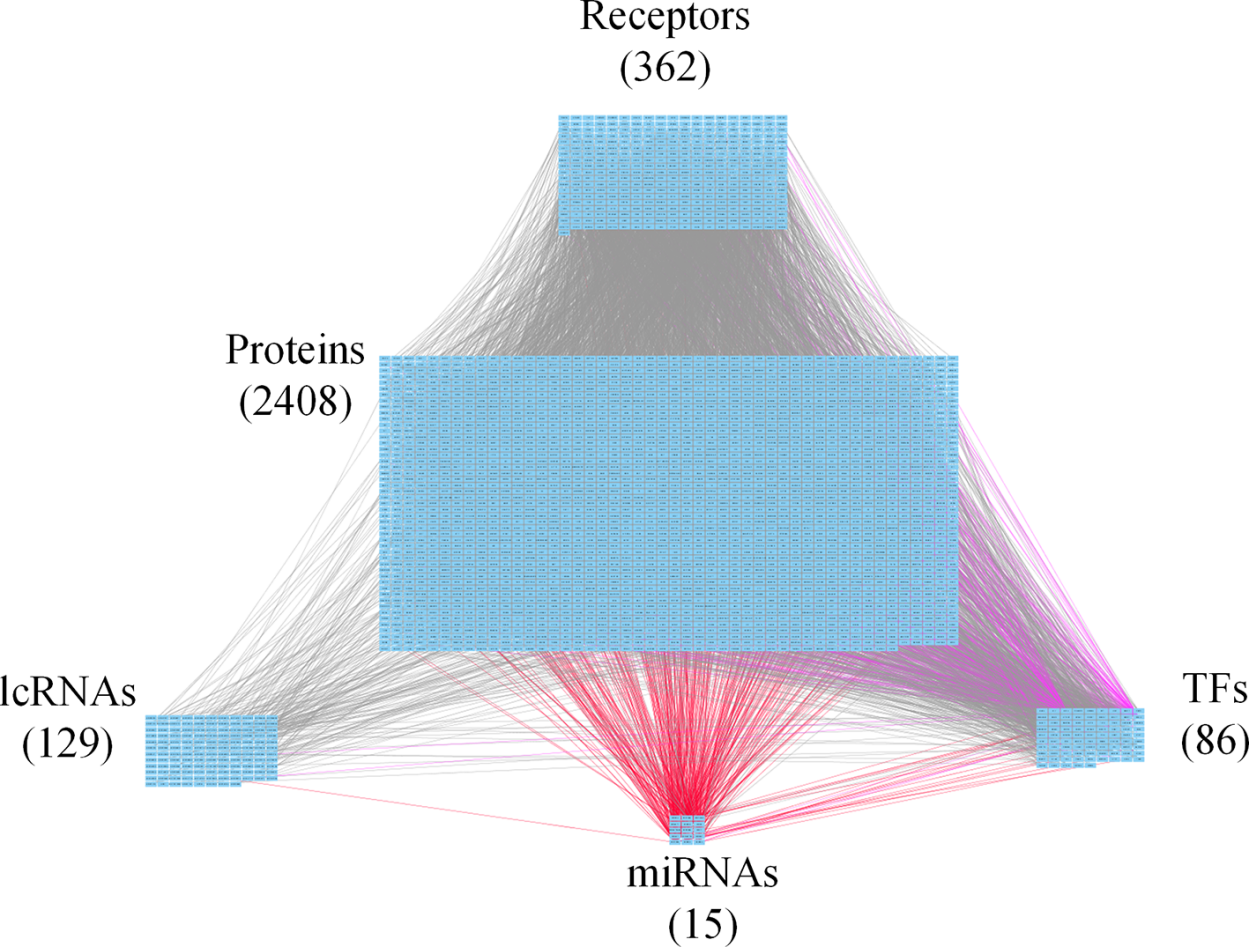
Core genome-wide genetic and epigenetic network (GWGEN) of mid-stage colorectal cancer (CRC) cells. The core GWGEN of mid-stage colon cancer was extracted from the corresponding real GWGEN by applying the principal network projection (PNP) method. Among the core GWGEN of mid-stage CRC, there are 362 receptors, 2,408 proteins, 129 lncRNAs, 15 miRNAs, and 86 transcription factors (TFs). The gray lines represent edges in protein-protein interaction network (PPIN); the purple lines represent edges in gene regulatory network (GRN); the red lines represent miRNA regulations.

**Figure 4:**
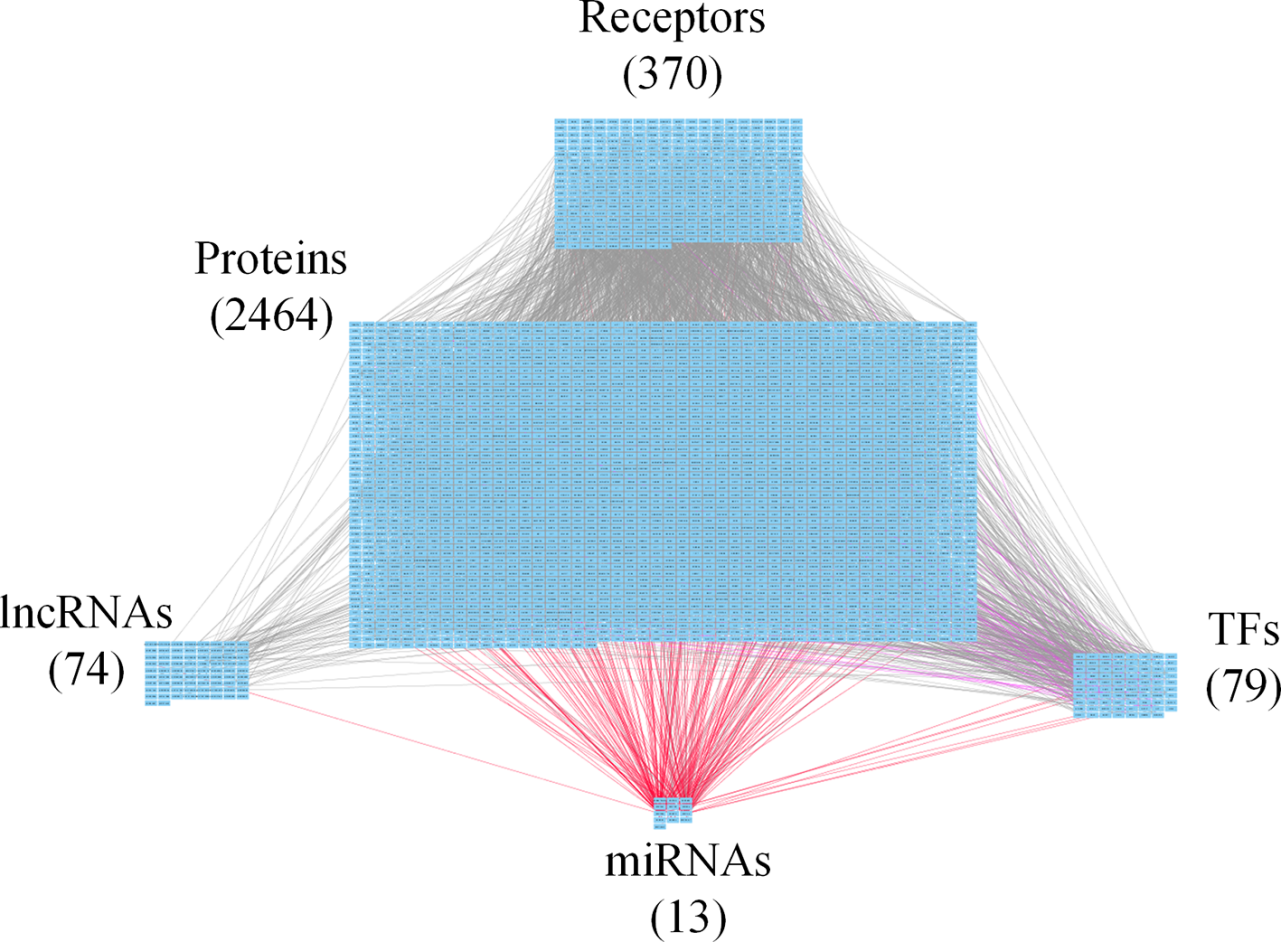
Core genome-wide genetic and epigenetic network (GWGEN) of late-stage colorectal cancer (CRC) cells. The core GWGEN of late-stage colon cancer was extracted from the corresponding real GWGEN by applying the principal network projection (PNP) method. Among the core GWGEN of late-stage CRC, there are 370 receptors, 2,464 proteins, 74 lncRNAs, 13 miRNAs, and 79 transcription factors (TFs). The gray lines represent edges in PPI; the purple lines represent edges in gene regulatory network (GRN); the red lines represent miRNA regulations.

**Figure 5:**
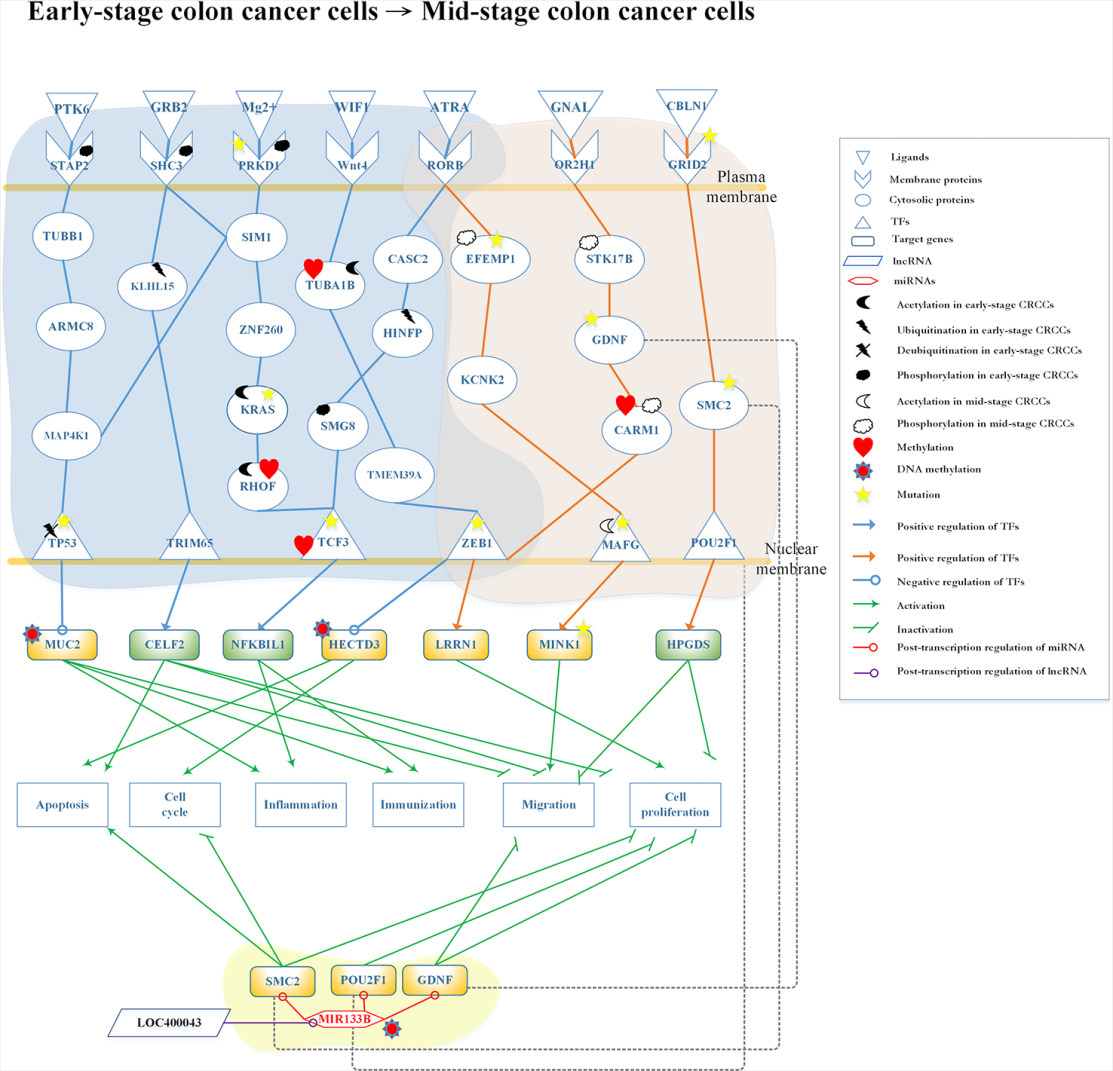
Differential core pathways for investigating the progression mechanism from early-stage colon cancer cells to mid-stage colon cancer cells. This figure shows the core pathways in the annotation of Kyoto Encyclopedia of Genes and Genomes (KEGG) pathways extracted from core genome-wide genetic and epigenetic networks (GWGENs) of early-stage colon cancer cells and mid-stage colon cancer cells. The blue background covers the core pathways in early-stage colon cancer cells; the orange background covers the core pathways in mid-stage colon cancer cells; the green background covers the regulation of MIR133B which is modified by DNA methylation to inhibit cell migration and proliferation in mid-stage colon cancer cells. The green rectangular represents downregulation. The yellow rectangular represents upregulation. The blue lines represent the pathways in early-stage colon cancer cells; the orange lines represent the pathways in mid-stage colon cancer cells; the gray dash lines represent translocation; the green lines with an arrow head stand for activation of function; the green lines with a bar head stand for repressing function; the blue and orange lines with an arrowhead and circle head denote activation and repression of function, respectively; the red lines with a circle mean repression of function; and the yellow stars refer to gene involvement in mutation.

**Figure 6:**
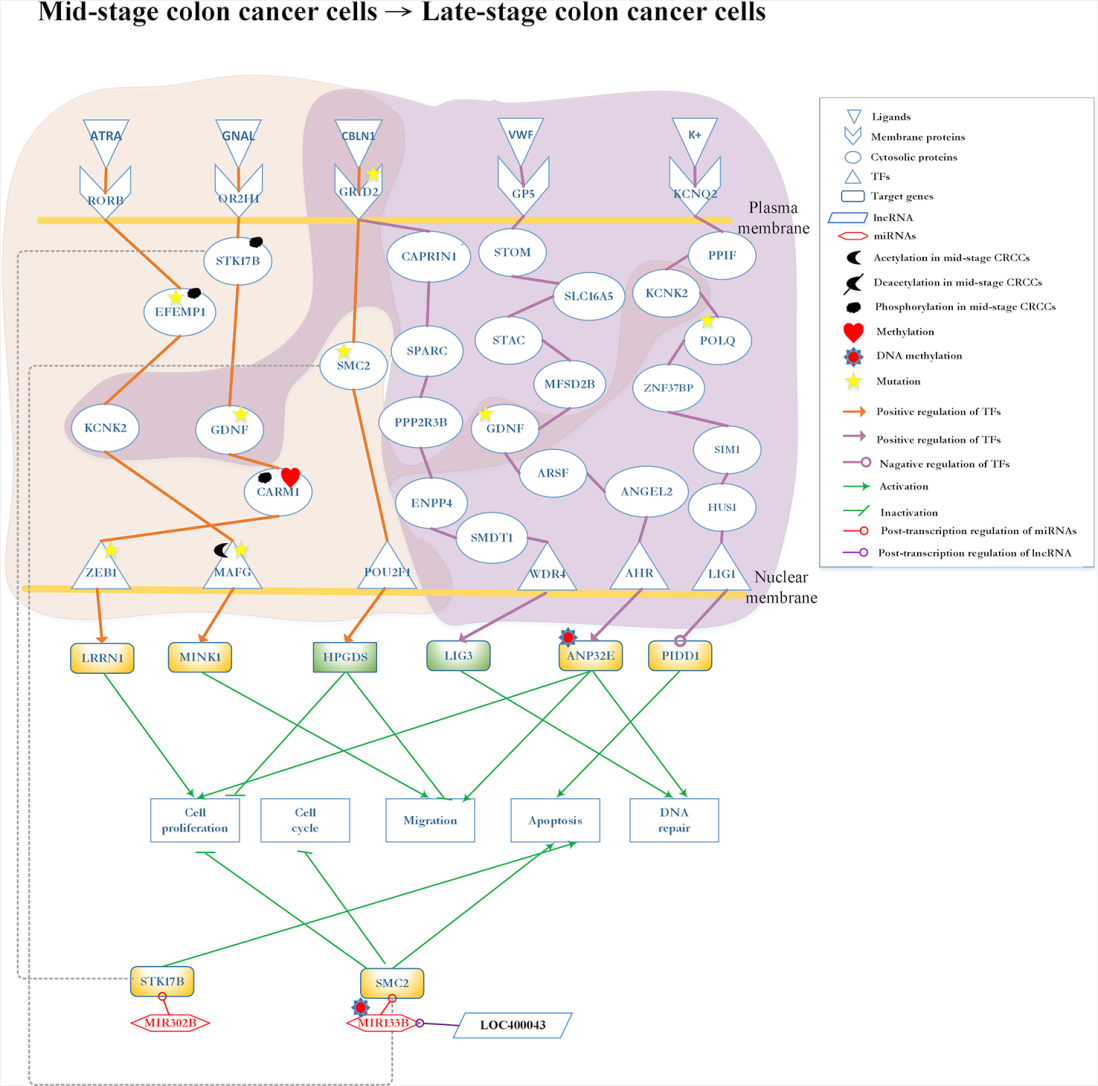
Differential core pathways for investigating the progression mechanism between mid-stage colon cancer cells and late-stage colon cancer cells. This figure shows the core pathways in the annotation of Kyoto Encyclopedia of Genes and Genomes (KEGG) pathways extracted from the core genome-wide genetic and epigenetic networks (GWGENs) of mid-stage colon cancer cells and late-stage colon cancer cells. The orange background covers the molecular mechanism in mid-stage colon cancer cells; the purple background covers the core pathways in late-stage colon cancer cells. The green rectangular represents downregulation. The yellow rectangular represents upregulation. The orange lines represent the pathways in mid-stage colon cancer cells; the purple lines represent the pathways in late-stage colon cancer cells; the grey dash lines represent translocation; the green lines with an arrow head stand for activation of function; the green lines with a bar head stand for repression of function; the orange and purple lines with an arrowhead and circle head denote activation and repression of function, respectively; the red lines with a circle mean repression of function; and the yellow stars refer to gene involvement in mutation.

To identify essential biomarkers and find potential multiple-molecule drug to prevent the progression of CRC, the flowchart is given in **Figure 1**. At first, we constructed the candidate GWGEN by mining big databases, including Database of Interacting Proteins (DIP), Biomolecular Interaction Network Database (BIND), Biological General Repository for Interaction Datasets (BioGRID), IntAct, MINT, Integrated Transcription Factor Platform (ITFP), and Circuits DB2. The genome-wide microarray raw data were downloaded from the Gene Expression Omnibus database (https://www.ncbi.nlm.nih.gov/geo/query/acc.cgi), including 290 primary colorectal tumor samples (GSE14333). For the convenience of analysis, we merged Dukes’ stages A and B into the early stage. Dukes’ stages C and D are considered as the mid stage and late stage, respectively (Jorissen et al., 2009). We separated the data in three stages: early stage (137 samples), mid stage (92 samples), and late stage (61 samples). Subsequently, we used the gene symbols of human gene information data from the National Center for Biotechnology Information (NCBI) website as standard human gene names. Secondly, with the help of above mentioned three stages of microarray data, we used system identification and model selection approaches to eliminate the false positives in the candidate GWGEN and obtain real GWGENs. Afterwards, since it was still too complicated to analyze the real GWGENs, the PNP method was applied to get core GWGENs shown as **Figures 2**–**4**. Moreover, based on the projection values, the core pathways shown as **Figures 5** and **6** in respect of KEGG pathways could be extracted out from the core GWGENs. Eventually, we suggested two potential multiple-molecule drugs to reverse the identified abnormal signature to avoid the progression of CRC by querying CMap.

### Construction of the Systematic Models for the GWGENs

For the PPIN, the expression levels of the *i*_*th*_ protein and its *h*_*th*_ interaction protein in the *n*th sample, denoted by *q*_*i*_ [*n*] and *ph*[*n*], respectively, could be

(1) $$q_{i}[n]=\sum_{\begin{array}{c} h=1\\ h\neq i \end{array}}^{H_{i}}a_{ih}q_{i}[n]q_{h}[n]+\beta_{i,PPIN}+\varepsilon_{i,PPIN}[n],\text{ for }i=1,\ldots ,I,n=1,\ldots ,N,$$

where *a*_*ih*_ is the interaction ability between the *i-th* protein and its *h*_*th*_ interactive protein; *Hi* indicates the total number of proteins interacting with the *i*_*th*_ protein; *β*_*i*, *PPIN*_ denotes the basal level of the *i*_*th*_ protein expression; *ε*_*i*,*PPIN*_ [*n*] represents the stochastic noise owing to the modeling residue and measurement noise in the *i*_*th*_ protein; *I* is the number of proteins; and *N* is the number of samples.

For the GRN, the expression level of a gene is regulated by its regulatory TFs/proteins, lncRNAs and *w*_*th*_ miRNAs could be described by the following gene regulatory equation:

(2) $$z_{j}[n]=\sum_{u=1}^{U_{j}}b_{ju}p_{u}[n]+\sum_{v=1}^{V_{j}}c_{jv}x_{v}[n]-\sum_{w=1}^{W_{j}}d_{\text{jw}}z_{j}[n]r_{w}[n]+\beta_{j,GRN}+\epsilon_{j,GRN}[n],$$

for j=1, …, *J*, n=1, …, *N*.

where *z*_*j*_ [*n*] is the expression level of the *j*_*th*_ gene; *p*_*u*_ [*n*], *x*_*v*_ [*n*] and *r*_*w*_ [*n*] denote the expressions of the *u*_*th*_ TF/protein, the *v*_*th*_ lncRNA and the *w*_*th*_ miRNA, respectively; *d*_*th*_≥ 0 represents the posttranscriptional regulatory ability of the *w*_*th*_ miRNA to inhibit the *j*_*th*_ gene; *b*_*ju*_ denotes the transcription regulatory ability from the *u*_*th*_ TF to the *j*_*th*_ gene; *Uj* indicates the total number of TFs binding to the *j*_*th*_ gene; *cjv* is the transcription regulatory ability from the *v*_*th*_ lncRNA to the *j*_*th*_ gene; *Vj* denotes the total number of lncRNAs binding to the *j*_*th*_ gene; *Wj* is the total number of miRNAs inhibiting the *j*_*th*_ gene; *βj*,*GRN* indicates the basal level of the *j*_*th*_ gene expression; *εj*,*GRN*[*n*] represents the stochastic noise owing to the modeling residue and measurement noise in the *j*_*th*_ gene; *J* is the total number of genes; and *N* denotes the number of samples (i.e., patients).

For the lncRNA regulatory network (LRN), the expression level of an lncRNA is regulated by its regulatory TFs/proteins, lncRNAs, and miRNAs is described by the following regulatory equation:

(3) $$f_{k}[n]=\sum_{u=1}^{U_{k}}e_{ku}p_{u}[n]+\sum_{v=1}^{V_{k}}t_{kv}x_{v}[n]-\sum_{w=1}^{{\text{W}}_{k}}r_{kw}f_{k}[n]r_{w}[n]+\beta_{k,LRN}+\varepsilon_{k,LRN}[n],$$

for k=1, …, K, n=1, …, N.

where f*k*[*n*] is the expression level of the *k*_*th*_ lncRNA; *pu*[*n*], *xv*[*n*], and *rw*[*n*] are the expression levels of regulatory TFs/proteins, lncRNAs and miRNAs, respectively; *rkw*≥ 0 denotes the posttranscriptional regulatory ability of the *w*_*th*_ miRNA to inhibit the *k*_*th*_ lncRNA; *eku* denotes the transcription regulatory ability from the *u*_*th*_ TF to the *k*_*th*_ lncRNA; *Uk* indicates the total number of TFs binding to the *k*_*th*_ lncRNA; *tkv* is the transcription regulatory ability from the *v*_*th*_ lncRNA to the *k*_*th*_ lncRNA; *Vk* denotes the total number of lncRNAs binding to the *k*th lncRNA; *Wk* is the total number of miRNAs inhibiting the *k*_*th*_ lncRNA; *βk*,*LRN* indicates the basal level of the *k*th lncRNA expression; *εk*,*LRN*[*n*] represents the stochastic noise owing to the modeling residue and measurement noise in the *k*_*th*_ lncRNA; and *K* is the total number of lncRNAs.

For the miRNA regulatory network (MRN), the expression level of a miRNA is regulated by its regulatory TFs/proteins, lncRNAs, and miRNAs could be described by the following regulatory equation:

(4) $$b_{t}[n]=\sum_{u=1}^{U_{t}}y_{tu}p_{u}[n]+\sum_{v=1}^{V_{t}}h_{tv}x_{v}[n]-\overset{W_{t}}{\underset{w=1}{\sum }}n_{tw}b_{t}\left[n\right]r_{w}\left[n\right]+\beta_{t,MRN}+\varepsilon_{t,MRN}\left[n\right],$$

for *t* = 1,…, *T*, *n* = 1,…, *N*,

where b*t*[*n*] is the expression level of the *t*_*th*_ miRNA; *pu*[*n*], *xv*[*n*], and *rw*[*n*] are the expression levels of regulatory TFs/proteins, lncRNAs, and miRNAs, respectively; n*tw*≥ 0 denotes the posttranscriptional regulatory ability of the *w*_*th*_ miRNA to inhibit the *t*_*th*_ miRNA; *ytu* denotes the transcription regulatory ability from the *u*_*th*_ TF to the *t*_*th*_ miRNA; *Ut* indicates the total number of TFs binding to the *t*_*th*_ miRNA; *htv* is the transcription regulatory ability from the *v*_*th*_ lncRNA to the *t*_*th*_ miRNA; *Vt* denotes the total number of lncRNAs binding to the *t*_*th*_ miRNA; *Wt* is the total number of miRNAs inhibiting the *t*_*th*_ miRNA; *βt*,*MRN* indicates the basal level of the *t*_*th*_ miRNA expression; *εt*,*MRN*[*n*] represents the stochastic noise owing to the modeling residue and measurement noise in the *t*_*th*_ miRNA; and *T* is the total number of miRNAs. Accordingly, we considered epigenetic modifications, such as acetylation, methylation, ubiquitination, and phosphorylation. These epigenetic modifications can contribute to change in the basal level indicated in equations (1–4) when compared in different stages.

### System Identification of Candidate GWGEN *via* Genome-Wide Microarray Data

To identify the precise parameters of the PPIN in equation (1) through the system identification method, equation (1) can be represented by the linear regression form as shown below:

(5) $$q_{i}[n]=\left[q_{i}[n]q_{1}[n]\;q_{i}[n]q_{2}[n]\cdots q_{i}[n]q_{H_{i}}[n]1\right]\cdot \left[\begin{array}{l} a_{i1}\\ a_{i2}\\ \vdots \\ a_{iH_{i}}\\ \beta_{i,PPIN} \end{array}\right]+\varepsilon_{i,PPIN}[n]$$

$$\equiv \phi_{i,PPIN}^{T}[n]\cdot \theta_{i,PPIN}+\varepsilon_{i,PPIN}[n],\text{ for }i=1,\ldots ,I,$$

where $\phi_{i,PPIN}^{T}[n]$ indicates the regression vector obtained from the corresponding microarray expression data, and *θ*_*i*,*PPIN*_ represents the unknown parameter vector to be estimated for the *i*_*th*_ protein in PPIN. The equation (5) of the *i*_*th*_ protein can be augmented for *N* samples as the following form:

(6) $$\left[\begin{array}{l} q_{i}[1]\\ \vdots \\ q_{i}[N] \end{array}\right]=\left[\begin{array}{l} \phi_{i,PPIN}^{T}[1]\\ \vdots \\ \phi_{i,PPIN}^{T}[N]\; \end{array}\right]\cdot \theta_{i,PPIN}+\left[\begin{array}{l} \varepsilon_{i,PPIN}[1]\\ \vdots \\ \varepsilon_{i,PPIN}[N] \end{array}\right],\text{ for }i=1,\ldots ,I,$$

which could be simply described as:

(7) $${\text{Q}}_{i}=\Phi_{i,PPIN}\cdot \theta_{i,PPIN}+\Xi_{i,PPIN}$$

where

$${\text{Q}}_{i}=\left[\begin{array}{c} q_{i}[1]\\ \vdots \\ q_{i}[N] \end{array}\right],\;\Phi_{i,PPIN}=\left[\begin{array}{c} \phi_{i,PPIN}^{T}[1]\\ \vdots \\ \phi_{i,PPIN}^{T}[N] \end{array}\right],\;\Xi_{i,PPIN}=\left[\begin{array}{c} \varepsilon_{i,PPIN}[1]\\ \vdots \\ \varepsilon_{i,PPIN}[N] \end{array}\right].$$

Therefore, by applying the following least-squares estimation problem, we could estimate the interactive parameters in the vector ${\hat{\theta }}_{i,PPIN}$:

(8) $${\hat{\theta }}_{i,PPIN}=\underset{\theta_{i,PPIN}}{\min}\frac{1}{2}{\left\|\Phi_{i,PPIN}\cdot \theta_{i,PPIN}-Q_{i}\right\|}_{2}^{2}.$$

By solving the above optimization problem (8), we can obtain the interactive parameters in the PPIN interactive equation (1).

The linear regression form of the gene regulatory equations in (2) of GEN could be described as shown below:

(9) $$z_{j}[n]=[p_{1}[n]\;\cdots p_{U_{j}}[n]x_{1}[n]\cdots x_{V_{j}}[n]\;z_{j}[n]r_{1}[n]\cdots z_{j}[n]r_{W_{j}}[n]]\cdot \left[\begin{array}{l} b_{j1}\\ \vdots \\ b_{jU_{j}}\\ c_{j1}\\ \vdots \\ c_{jV_{j}}\\ -d_{j1}\\ \vdots \\ -d_{jW_{j}}\\ \beta_{j,GRN} \end{array}\right]+\varepsilon_{j,GRN}[n]$$

$$\equiv \phi_{j.GRN}^{T}[n]\cdot \theta_{j,GRN}+\varepsilon_{j,GRN}[n],\text{ for }j=1,\ldots ,J,$$

where $\phi_{j.GRN}^{T}[n]$ indicates the regression vector obtained from the corresponding microarray expression data, and *θ*_*j*,*GRN*_ represents the unknown parameter vector to be estimated for the *j*_*th*_ gene in GRN. The equation (9) of the *j*_*th*_ gene could be augmented for *N* samples as the following form:

(10) $$\left[\begin{array}{l} \;z_{i}[1]\\ \;\vdots \\ z_{i}[N]\; \end{array}\right]=\left[\begin{array}{l} \;\phi_{j,GRN}^{T}[1]\\ \;\vdots \\ \phi_{j,GRN}^{T}[N]\; \end{array}\right]\cdot \theta_{j,GRN}+\left[\begin{array}{l} \;\varepsilon_{j,GRN}[1]\\ \;\vdots \\ \varepsilon_{j,GRN}[N]\; \end{array}\right],$$

for *j*=1, …, *J*.

which could be simply described as:

(11) $$Z_{j}=\Phi_{j,GRN}\cdot \theta_{j,GRN}+\Xi_{j,GRN}$$

where

$$Z_{j}=\left[\begin{array}{c} z_{i}[1]\\ \vdots \\ z_{i}[N] \end{array}\right],\;{\text{Φ}}_{j,GRN}=\left[\begin{array}{c} \phi_{j,GRN}^{T}[1]\\ \vdots \\ \phi_{j,GRN}^{T}[N] \end{array}\right]{\text{Ξ}}_{j,GRN}=\left[\begin{array}{c} \varepsilon_{j,GRN}[1]\\ \vdots \\ \varepsilon_{j,GRN}[N] \end{array}\right]$$

Therefore, by solving the following constrained least-squares estimation problem, we could estimate the regulatory parameters in the vector ${\hat{\theta }}_{j,GRN}$:

$${\hat{\theta }}_{\text{j,GRN}}=\underset{\theta_{j,GRN}}{\min}\frac{1}{2}{\left\|\Phi_{j,GRN}\cdot \theta_{j,GRN}-Z_{j}\right\|}_{2}^{2}$$

subject to

(12) $$\overset{U_{j}+V_{j}}{\overset{︷}{[\begin{array}{ccc} 0 & \cdots & 0\\ \vdots & \ddots & \vdots \\ 0 & \cdots & 0 \end{array}}}\;\overset{W_{j}}{\overset{︷}{\begin{array}{cccc} 1 & \cdots & 0 & 0\\ \vdots & \ddots & \vdots & \vdots \\ 0 & \cdots & 1 & 0 \end{array}}}\begin{array}{c} 0\\ \vdots \\ 0 \end{array}]\cdot \theta_{j,GRN}\leq \left[\begin{array}{c} 0\\ \vdots \\ 0 \end{array}\right]$$

Simultaneously, the miRNA repression parameter *-djw* is guaranteed to be nonpositive (i.e. *−djw* ≤ 0, for *w*=1, 2, …, *Wj*.).

Similarly, the regulatory equations of LRN in (3) could be described below:

$$f_{k}[n]=[p_{1}[n]\;\cdots \;p_{U_{k}}[n]\;x_{1}[n]\;\cdots \;x_{V_{k}}[n]\;$$

$$f_{k}[n]r_{1}[n]\;\cdots \;f_{k}[n]r_{W_{k}}[n]\;]\cdot$$

$$\left[\begin{array}{l} e_{k1}\\ \;\vdots \\ e_{kU_{k}}\\ t_{k1}\\ \;\vdots \\ t_{kV_{k}}\\ -r_{k1}\\ \;\vdots \\ -r_{kW_{k}}\\ \beta_{j,LRN} \end{array}\right]+\varepsilon_{k,LRN}[n]$$

(13) $$\equiv \phi_{k,LRN}^{T}[n]\cdot \theta_{k,LRN}+\varepsilon_{k,LRN}[n],$$

for *k*=1, …, *K*,

where $\phi_{k,LRN}^{T}[n]$ indicates the regression vector which can be obtained from corresponding microarray expression data, and *θ*_*k*,*LRN*_ represents the unknown parameter vector to be estimated for the *k*th lncRNA in LRN. The equation (13) of the *k*_*th*_ lncRNA could be augmented for *N* samples of corresponding microarray expression data as the following form:

(14) $$\left[\begin{array}{l} \;f_{k}[1]\\ \;\vdots \\ f_{k}[N]\; \end{array}\right]=\left[\begin{array}{l} \;\phi_{k,LRN}^{T}[1]\\ \;\vdots \\ \phi_{k,LRN}^{T}[N]\; \end{array}\right]\cdot \theta_{k,LRN}+\left[\begin{array}{l} \;\varepsilon_{k,LRN}[1]\\ \;\vdots \\ \varepsilon_{k,LRN}[N]\; \end{array}\right],$$

for *k*=1, …, *K*,

which could be simply described as:

(15) $$L_{k}=\Phi_{k,LRN}\cdot \theta_{k,LRN}+\Xi_{k,LRN}$$

where

$$L_{k}=\left[\begin{array}{c} f_{k}[1]\\ \vdots \\ f_{k}[N] \end{array}\right],\;\Phi_{k,LRN}=\left[\begin{array}{c} \phi_{k,LRN}^{T}[1]\\ \vdots \\ \phi_{k,LRN}^{T}[N] \end{array}\right],\;\Xi_{k,LRN}=\left[\begin{array}{c} \varepsilon_{k,LRN}[1]\\ \vdots \\ \varepsilon_{k,LRN}[N] \end{array}\right].$$

Therefore, by applying the following constrained least-squares estimation problem, we could estimate the regulatory parameters in the vector ${\hat{\theta }}_{k,LRN}$:

$${\hat{\theta }}_{k,LRN}=\underset{\theta_{k,LRN}}{\min}\frac{1}{2}{\left\|{\text{Φ}}_{k,LRN}\cdot \theta_{k,LRN}-L_{k}\right\|}_{2}^{2}$$

subject to

(16) $$\overset{U_{k}+V_{k}}{\overset{︷}{[\begin{array}{l} \begin{array}{ccc} 0 & \cdots & 0 \end{array}\\ \begin{array}{ccc} \vdots & \ddots & \vdots \end{array}\\ \begin{array}{ccc} 0 & \cdots & 0 \end{array} \end{array}}}\begin{array}{l} \overset{{\text{W}}_{k}}{\overset{︷}{\begin{array}{cccc} 1 & \cdots & 0 & 0 \end{array}}}\\ \begin{array}{cccc} \vdots & \ddots & \vdots & \vdots \end{array}\;\\ \begin{array}{cccc} 0 & \cdots & 1 & 0 \end{array} \end{array}\begin{array}{l} 0\\ \vdots \\ 0 \end{array}]\cdot \theta_{k,LRN}\leq \left[\begin{array}{l} 0\\ \;\vdots \\ 0 \end{array}\right].$$

By solving the above constrained optimization problem (16), we can obtain the parameters in the LRN regulatory equation (3). Simultaneously, the miRNA repression *−qkw* is guaranteed to be nonpositive, i.e., *−qkw* ≤ 0, for *w*=1, 2, …, *Wk*.

Finally, the linear regression form of the MRN regulatory equation (4) could be described as shown below:

$$b_{t}[n]=[p_{1}[n]\cdots p_{U_{t}}[n]x_{1}[n]\cdots x_{V_{t}}[n]\;b_{t}[n]r_{1}[n]\cdots b_{t}[n]r_{W_{t}}[n]\;]\cdot \left[\begin{array}{l} y_{t1}\\ \vdots \\ y_{sU_{t}}\\ h_{t1}\\ \vdots \\ h_{tV_{t}}\\ -n_{t1}\\ \vdots \\ -n_{tW_{t}}\\ \beta_{t,MRN} \end{array}\right]+\varepsilon_{t,MRN}[n]$$

(17) $$\equiv \phi_{t,MRN}^{T}[n]\cdot \theta_{t,MRN}+\varepsilon_{t,MRN}[n],\;\text{ for }t=1,\ldots ,T,$$

where $\phi_{t,MRN}^{T}[n]$ indicates the regression vector which can be obtained for *N* samples of corresponding microarray expression data, and *θ*_*t*,*MRN*_ represents the unknown parameter vector to be estimated for the *t*_*th*_ miRNA in MRN.

The regulatory equation (17) of the *t*_*th*_ miRNA could be augmented for *N* samples of corresponding microarray expression data as the following form:

(18) $$\left[\begin{array}{l} \;b_{t}[1]\\ \;\vdots \\ b_{t}[N]\; \end{array}\right]=\left[\begin{array}{l} \;\phi_{t,MRN}^{T}[1]\\ \;\vdots \\ \phi_{t,MRN}^{T}[N]\; \end{array}\right]\cdot \theta_{t,MRN}+\left[\begin{array}{l} \;\varepsilon_{t,MRN}[1]\\ \;\vdots \\ \varepsilon_{t,MRN}[N]\; \end{array}\right],\text{for }t=1,\ldots ,T,$$

which could be simply described as:

(19) $$M_{t}=\Phi_{t,MRN}\cdot \theta_{t,MRN}+\Xi_{t,MRN}$$

where

$$M_{t}=\left[\begin{array}{c} b_{t}[1]\\ \vdots \\ b_{t}[N] \end{array}\right],\;\Phi_{t,MRN}=[\begin{array}{c} \phi_{t,MRN}^{T}[1]\\ \vdots \\ \phi_{t,MRN}^{T}[N] \end{array}],\;\Xi_{t,MRN}=\left[\begin{array}{c} \varepsilon_{t,MRN}[1]\\ \vdots \\ \varepsilon_{t,MRN}[N] \end{array}\right]$$

Therefore, by applying the following constrained least-squares estimation problem, we could estimate the regulatory parameters in the vector ${\hat{\theta }}_{t,MRN}$:

$${\hat{\theta }}_{t,MRN}=\underset{\theta_{t,MRN}}{\min}\frac{1}{2}{\left\|\Phi_{t,MRN}\cdot \theta_{t,MRN}-M_{t}\right\|}_{2}^{2}$$

subject to

(20) $$\overset{U_{t}+V_{t}}{\overset{︷}{[\begin{array}{l} \begin{array}{ccc} 0 & \cdots & 0 \end{array}\\ \begin{array}{ccc} \vdots & \ddots & \vdots \end{array}\\ \begin{array}{ccc} 0 & \cdots & 0 \end{array} \end{array}}}\begin{array}{l} \overset{{\text{W}}_{t}}{\overset{︷}{\begin{array}{cccc} 1 & \cdots & 0 & 0 \end{array}}}\\ \begin{array}{cccc} \vdots & \ddots & \vdots & \vdots \end{array}\;\\ \begin{array}{cccc} 0 & \cdots & 1 & 0 \end{array} \end{array}\begin{array}{l} 0\\ \vdots \\ 0 \end{array}]\cdot \theta_{t,MRN}\leq \left[\begin{array}{l} 0\\ \vdots \\ 0 \end{array}\right]$$

By solving the above constrained optimization problem (20), we can obtain the parameters in the MRN regulatory equation (4). Simultaneously, the miRNA repression *−ztw* is guaranteed to be nonpositive, i.e., *−ztw* ≤ 0, for *w*=1, 2, …, *Wt*.

### System Order Detection Scheme for Pruning the False Positives of the Candidate Network to Obtain the Real GWGENs of Early-Stage to Late-Stage CRC

Big database mining is associated with many false positives in the constructed candidate GWGEN. Therefore, we pruned the false positives in the candidate GWGEN to obtain the real GWGENs for the three stages of CRC using a system order detection scheme.

For the PPIN model in (7), the Akaike information criterion (AIC) for detecting the number of interactions of the *i*_*th*_ protein could be defined using the following equation:

(21) $${\text{AIC}}_{i}(H_{i})=\log\left(\frac{1}{N}{\left({\text{Q}}_{i}-\Phi_{i,PPIN}\cdot {\hat{\theta }}_{i,PPIN}\right)}^{T}({\text{Q}}_{i}-\Phi_{i,PPIN}\cdot {\hat{\theta }}_{i,PPIN})\right)+\frac{2(H_{i}+1)}{N}$$

where ${\hat{\theta }}_{i,PPIN}$ indicates the estimated interaction parameter vector of the *i*_*th*_ protein by solving the equation,$\frac{1}{N}{\left({\text{Q}}_{i}-\Phi_{i,PPIN}\cdot {\hat{\theta }}_{i,PPIN}\right)}^{T}({\text{Q}}_{i}-\Phi_{i,PPIN}\cdot {\hat{\theta }}_{i,PPIN})$ is the estimated residual error. A decrease in the system interaction number (order) *Hi* will result in an increase in the corresponding estimated residual error. In contrast, attempts to minimize the estimated residual error, will result in an increase in the corresponding system order. Hence, we need to trade-off the system order and estimated residual error to determine the minimum value of the *AICi* for the real system interaction order *Hi*, i.e., the real system order ${\text{H}}_{i}^{*}$ of protein *i* could minimize AIC_*i*_(*H*_*i*_) in (21). Therefore, the insignificant protein interactions out of system order${\text{H}}_{i}^{*}$ should be pruned from the interactions of the candidate PPIN. Gradually (one protein each time), we could prune the false positives of all proteins in the candidate PPIN to obtain the real PPIN using the AIC system order detection method. Similarly, for pruning false positives of the candidate GRN subnetwork in (11), the candidate LRN subnetwork in (15), and the candidate MRN subnetwork in (19), AICs for system order detection of the *j*_*th*_ gene, the *k*th lncRNA, and the *t*_*th*_ miRNA could be defined using the following equations, respectively:

(22) $$\begin{array}{l} {\text{AIC}}_{j}(U_{j},V_{j},W_{j})=\log\left(\frac{1}{N}{\left(Z_{j}-\Phi_{j,GRN}\cdot {\hat{\theta }}_{j,GRN}\right)}^{T}(Z_{j}-\Phi_{j.GRN}\cdot {\hat{\theta }}_{j,GRN})\right)\\ \;+\frac{2(U_{j}+V_{j}+W_{j}+1)}{N} \end{array}$$

(23) $$\begin{array}{l} {\text{AIC}}_{k}(U_{k},V_{k},W_{k})=\log\left(\frac{1}{N}{\left(L_{k}-\Phi_{k,LRN}\cdot {\hat{\theta }}_{k,LRN}\right)}^{T}(L_{k}-\Phi_{k,LRN}\cdot {\hat{\theta }}_{k,LRN})\right)\\ \;+\frac{2(U_{k}+V_{k}+W_{k}+1)}{N} \end{array}$$

(24) $$\begin{array}{l} {\text{AIC}}_{t}(U_{t},V_{t},W_{t})=\log\left(\frac{1}{N}{\left(M_{t}-\Phi_{t,MRN}\cdot {\hat{\theta }}_{t,MRN}\right)}^{T}(M_{t}-\Phi_{t,MRN}\cdot {\hat{\theta }}_{t,MRN})\right)\\ \;+\frac{2(U_{t}+V_{t}+W_{t}+1)}{N} \end{array}$$

where ${\hat{\theta }}_{j,GRN}$,${\hat{\theta }}_{k,LRN}$,${\hat{\theta }}_{t,MRN}$ indicate the estimated parameter vector of the *j*_*th*_ gene, the *k*_*th*_ lncRNA, and the *t*_*th*_ miRNA by solving the equations (11), (15), and (19), respectively; $\frac{1}{N}{\left(Z_{j}-\Phi_{j,GRN}\cdot {\hat{\theta }}_{j,GRN}\right)}^{T}(Z_{j}-\Phi_{j.GRN}\cdot {\hat{\theta }}_{j,GRN})$, $\frac{1}{N}{\left(L_{k}-\Phi_{k,LRN}\cdot {\hat{\theta }}_{k,LRN}\right)}^{T}(L_{k}-\Phi_{k,LRN}\cdot {\hat{\theta }}_{k,LRN})$, and $\frac{1}{N}{\left(M_{t}-\Phi_{t,MRN}\cdot {\hat{\theta }}_{t,MRN}\right)}^{T}(M_{t}-\Phi_{t,MRN}\cdot {\hat{\theta }}_{t,MRN})$ are the estimated residual error, respectively. In (22), (23), and (24), we could select $U_{j}^{*}$,$V_{j}^{*}$,$W_{j}^{*}$,$U_{k}^{*}$,$V_{k}^{*}$,$W_{k}^{*}$, and $U_{t}^{*}$,$V_{t}^{*}$,$W_{t}^{*}$, to minimize AIC_*j*_(*U*_*j*_,*V*_*j*_,*W*_*j*_),AIC_*k*_(*U*_*k*_,*V*_*k*_,*W*_*k*_), and AIC_*t*_(*U*_*t*_,*V*_*t*_,*W*_*t*_), respectively. Then, $U_{j}^{*}$,$V_{j}^{*}$, and $W_{j}^{*}$ would be the real numbers of the regulatory TFs, lncRNAs, and miRNAs of the *j*_*th*_ gene, respectively; $U_{k}^{*}$,$V_{k}^{*}$, and $W_{k}^{*}$ would be the real numbers of the regulatory TFs, lncRNAs, and miRNAs of the *k*_*th*_ lncRNA, respectively; $U_{t}^{*}$,$V_{t}^{*}$ and $W_{t}^{*}$ would be the real numbers of the regulatory TFs, lncRNAs, and miRNAs of the *t*_*th*_ miRNA, respectively. The insignificant regulatory TFs lncRNAs, and miRNAs out of $U_{j}^{*}$,$V_{j}^{*}$, and ${\text{W}}_{j}^{*}$ in the *i*th gene, $U_{k}^{*}$,$V_{k}^{*}$, and $W_{k}^{*}$ in the *j*th lncRNA, $U_{t}^{*}$,${\text{V}}_{t}^{*}$, and $W_{t}^{*}$ in the *t*_*th*_ miRNA are considered false positives in the candidate GWGENs. By gradually pruning the false positives (i.e., one gene, one lncRNA, and one miRNA each time), we could obtain the real GWGENs at every stage of CRC. The GWGENs of early-stage, mid-stage, and late-stage CRC, constructed using the proposed systems biology method, are illustrated in **Figures S1**, **S2**, and **S3**, respectively.

### Extract of the Core Network From Real GWGENs by Applying the PNP Method

It is difficult to directly investigate the genetic and epigenetic mechanisms of colon carcinogenesis using the real GWGENs owing to their complexity. Prior to using the PNP method for the extraction of the core GWGENs from the real GWGENs, we initially established a network matrix P, including all the estimated parameters of edges in the real GWGENs. The network matrix P of real GWGEN is shown below:

(25) P=[a^11⋯a^1I⋮a^ih⋮a^I1⋯a^IH0⋯0⋮0⋮0⋯00⋯0⋮0⋮0⋯0b^11⋯b^1U⋮b^ju⋮b^J1⋯b^JUc^11⋯c^1V⋮c^kr⋮c^J1⋯c^JV−d^11⋯−γ^1w⋮−d^kx⋮−d^J1⋯−d^Jwe^11⋯e^1U⋮e^ku⋮e^K1⋯e^KUt^11⋯t^1V⋮t^kv⋮t^K1⋯t^KV−r^11⋯−r^1W⋮−r^kw⋮−r^K1⋯−r^KWy^11⋯y^1U⋮y^tu⋮y^T1⋯y^TUh^11⋯h^1V⋮h^tv⋮h^T1⋯h^TV−n^11⋯−n^1W⋮−n^tw⋮−n^T1⋯−n^TW],∈ℝ(I+J+K+T)×(U+V+W)

where α^ih could be obtained in ${\hat{\theta }}_{i,PPIN}$ by solving the equation (8) and pruning false positives through the AIC method in (21); ${\hat{b}}_{ju}$,${\hat{c}}_{jv}$,$-{\hat{d}}_{jw}$ could be obtained in ${\hat{\theta }}_{j,GRN}$ by solving the equation (12) and pruning false positives using the AIC method in (22); ${\hat{e}}_{ku}^{}$,${\hat{t}}_{kv}^{}$, and $-{\hat{r}}_{kw}^{}$ could be obtained in ${\hat{\theta }}_{k,LRN}$ by solving the equation (16) and pruning false positives *via* the AIC method in (23); ${\hat{y}}_{tu}^{}$,${\hat{h}}_{tv}^{}$, and$-{\hat{n}}_{tw}^{}$ could be obtained in ${\hat{\theta }}_{t,MRN}$ by solving the equation (20) and pruning false positives by the AIC method in (24); *U*, *V*, and *W* are the total number of TFs, lncRNAs, and miRNAs, respectively. The estimated interactive and regulative abilities of edges in real GWGEN are included in the network matrix P. If an edge does not exist in the GWGEN or has been pruned out through AIC, the corresponding parameter in network matrix P is padded with zero.

Subsequently, we extracted the core components of the GEN using the PNP method. The proposed PNP method is a principal network structure projection method based on principal singular value decomposition of the system matrix *P* in (25) as follows:

(26) $$P=\text{Q}\times D\times R^{T}$$

where unitary matrix ∈ *ℝ* ^(*I*+*J*+*K*+*T*)×(*U*+*V*+*W*)^; *R*∈ *ℝ* ^(*U*+*V*+*W*)×(*U*+*V*+*W*)^, and *D*=*diag*(*d*_1_,...,*d*_(*U*+*V*+*W*)_) is a diagonal matrix, which contains *U*+*V*+*W* singular values of P in a descending order, i.e., *d*_1_≥...≥ *d*_*U*+*V*+*W*_.

The eigen expression fraction *Em* is defined as the following energy normalization:

(27) $$E_{m}=\frac{d_{m}^{2}}{\sum_{m=1}^{U+V+W}d_{m}^{2}}.$$

By selecting the minimum *A* $\sum_{m=1}^{A}E_{m}\geq 0.85$, the *A* top singular vectors of matrix *P* contained 85% of the core network structure of the real GWGEN from the energy point of view. Next, the projection of *P* to the top *A* singular vectors of *N* is defined as follows:

(28) $$N_{R}\left(\omega ,\ell \right)=a_{\omega ,:}\times r_{:,\ell }$$

for *ω* = 1,…,*I* +* J* +*K* + *T and* ℓ = 1,…,*A*,

where *a*_*ω*,:_ represents the *ω*_*th*_ row vector of *P*; and *r*_:*ℓ*_ represents the $\ell_{\text{th}}$ column vectors of *N*.

Next, we defined the 2-norm projection value of each node, including proteins, genes, lncRNAs, and miRNAs in GWGENs to the top *A* right-singular vectors

(29) $$D_{R}(\omega )={\left[{\sum_{m=1}^{A}\left[N_{R}(\omega ,\ell )\right]}^{2}\right]}^{\frac{1}{2}}$$

for *ω* = 1,…, (*I * + *J *+ *K *+ *T*), *and ℓ* = 1,…,*A*,

where *D*_*R*_(*ω*) is the 2-norm projection value of the *ω*_*th*_ node of GWGEN on the 85% core network architecture. We selected important proteins from receptors to TFs and their associated miRNAs, lncRNAs, and genes to construct core GWGENs for the investigation of significant genetic and epigenetic mechanisms in early-stage, mid-stage, and late-stage CRC cells. Finally, the core GWGENs of early-stage to late-stage CRC are shown in **Figures 5** and **6**.

## Results

### Overview of Constructing Genome-Wide Genetic and Epigenetic Network for Early-Stage, Mid-Stage and Late-Stage CRC

In this study, to identify core GWGENs for three stages of CRC, we did big database mining to construct candidate PPI and candidate GRN. The candidate GWGEN consists of candidate PPI and candidate GRN. We applied reversed engineering and model selection approaches with corresponding early-stage, mid-stage, and late-stage of CRC microarray data to obtain real GWGENs shown in **Figures S1**, **S2**, and **S3**, respectively. The total number of nodes including receptors, proteins, lncRNAs, TFs, and miRNAs and edges of their interactions in candidate GWGEN and real GWGENs for three stages of CRC are shown in **Table 1**. It is noted that the number of nodes and edges decline a lot compared to those in real GWGENs. This phenomenon showed that the false positives were removed by system identification and model selection approaches. Afterwards, we utilized the PNP method, which could help to extract core GWGENs based on significant projection value of the node. The higher the projection value is, the more contribution made by the node in the core GWGEN. For the early-stage core GWGEN of CRC cells as shown in **Figure 2**, we identified 340 receptors, 2,455 proteins, 101 lncRNAs, 17 miRNAs, and 87 TFs. For the middle-stage core GWGEN of CRC cells as shown in **Figure 3**, we identified 362 receptors, 2,408 proteins, 129 lncRNAs, 15 miRNAs, and 86 TFs. For the late-stage core GWGEN of CRC cells as shown in **Figure 4**, 370 receptors, 2,464 proteins, 74 lncRNAs, 13 miRNAs, and 79 TFs are identified. The identified nodes in three stages of CRC could be found in **Supplementary Material**. Moreover, in order to be convenient for investigating the genetic and epigenetic molecular mechanisms of CRC, we denoted core signaling pathways in respect of KEGG pathways. The differential core signaling pathways were identified and carcinogenic mechanisms were found for early stage to mid stage and mid stage to late stage of CRC, shown in **Figures 5** and **6**, respectively. Consequently, according to our analytic results, we identified two groups of essential biomarkers reflecting abnormal gene expression signatures for CRC progression. By querying CMap, we suggested two multiple-molecule drugs for early stage to mid stage and mid stage to late stage of CRC, respectively.

**Table 1:** Number of edges and nodes in the candidate genome-wide genetic and epigenetic network (GWGEN) and identified real GWGENs in each stage of colorectal cancer.

	Candidate GWGEN	Early-stage real GWGEN	Mid-stage real GWGEN	Late-stage real GWGEN
TFs	2,221	461	517	522
TF-gene	84,426	8,633	8,183	8,102
TF-lncRNA	354	177	171	166
TF-miRNA	676	47	47	44
lncRNAs	261	236	233	224
lncRNA-gene	1,011	57	92	55
lncRNA-lncRNA	6	0	1	0
lncRNAs-miRNA	0	0	0	0
miRNAs	170	167	168	158
miRNA-gene	40,835	4,837	4,275	3,250
miRNA-lncRNA	134	19	19	13
miRNA-miRNA	1	0	0	1
Receptors	2,325	2,322	2,321	2,318
Proteins	14,749	14,594	14,537	14,364
PPIS	4,529,747	1,140,631	849,632	960,001
Total Nodes	19,726	17,780	17,776	17,586
Total Edges	4,657,190	1,154,382	862,430	971,632

### Core Pathways in Early-Stage to Mid-Stage CRC

The core differential pathways between early-stage and mid-stage CRC are shown in **Figure 5**. Our results demonstrated that the receptor signal transducing adaptor family member 2 (STAP2), which binds the ligand protein tyrosine kinase 6 (PTK6) (hypoxic microenvironment), interacts with tubulin beta 1 class VI (TUBB1) and transmits the signal through protein mitogen-activated protein kinase kinase kinase kinase 1 (MAP4K1) to TF tumor protein p53 (TP53). TP53 is modified by deubiquitination and downregulates the expression of target gene *MUC2*, which is also modified by DNA methylation. This epigenetic modification can be detected by the basal level. The ligand growth factor receptor bound protein 2 (GRB2) binds to the receptor SHC3 modified by phosphorylation and transmits to proteins kelch like family member 15 (KLHL15) and SIM1. Following the modification of KLHL15 by ubiquitination, the signal will be transmitted to TF tripartite motif containing 65 (TRIM65). In the meantime, TF TRIM65 upregulates *CUGBP Elav-like family member 2* (*CELF2*) to promote tumor apoptosis and inhibit cell migration and proliferation (Ramalingam et al., 2012).

The receptor protein kinase D1 (PRKD1), which is modified by phosphorylation and mutation, binds Mg2*+* and transmits the signal to SIM1, MAP4K1, and zinc finger protein 260 (ZNF260). ZNF260 transmits the signal to KRAS which is modified by mutation, and then transmits it to Ras homolog family member F (RHOF) modified by acetylation and methylation. Finally, the signal reaches the TF TCF3, which is modified by mutation and methylation. This results in the upregulation of the target gene *NFKB inhibitor like 1* (*NFKBIL1*), promoting immune response and inflammation (Atzei et al., 2010). The ligand WNT inhibitory factor 1 (WIF1) (signal transduction) binds to the receptor WNT4 and transmits the signal to TF zinc finger E-box binding homeobox 1 (ZEB1). ZEB1 appears in early-stage and mid-stage CRC cells through tubulin alpha 1b (TUBA1B), which is modified by acetylation and methylation, as well as transmembrane protein 39A (TMEM39A). ZEB1 modified by mutation downregulates *HECTD3*, which is also modified by DNA methylation. The aforementioned pathway controls the cell cycle and tumor apoptosis in early-stage CRC cells. Moreover, these epigenetic modifications can be detected by the basal level. The receptor RAR-related orphan receptor B (RORB), which is duplicated in early-stage and mid-stage CRC cells, binds ligand all-trans retinoic acid (ATRA) (apoptosis signal). Subsequently, it transmits the signal to TF TCF3 through cancer susceptibility 2 (CASC2), histone H4 transcription factor (HINFP) (modified by ubiquitination), and SMG8 (modified by phosphorylation) in early-stage CRC cells. TF TCF3 activates target gene *NFKBIL1* to promote inflammation and immune response.

In the mid-stage CRC cells, there are three core pathways transmitting the signals after the binding of three receptors with three ligands and involving several transduction proteins to reach three TFs. Notably, receptor RORB and TF ZEB1 also present in early-stage CRC cells. Firstly, the receptor RORB binds ligand ATRA (apoptosis signal) and then transmits the signal to EGF-containing fibulin extracellular matrix protein 1 (EFEMP1), (modified by phosphorylation and mutation) and potassium two pore domain channel subfamily K member 2 (KCNK2) to interact with TF MAF bZIP transcription factor G (MAFG) (modified by phosphorylation and mutation). Eventually, the target gene *MINK1* is upregulated, potentially leading to the migration of cancer cells to mid-stage CRC (Hu et al., 2004). Secondly, ligand G protein subunit alpha L (GNAL) (chemical stimulation) binds to the receptor olfactory receptor family 2 subfamily H member 1 (OR2H1) and transmits the signal through STK17B modified by phosphorylation, GDNF modified by mutation, and coactivator associated arginine methyltransferase 1 (CARM1) modified by acetylation and methylation to TF ZEB1. TF ZEB1 modified by mutation upregulates target gene LRRN1, which promotes proliferation in mid-stage CRC cells (Hossain et al., 2008). Thirdly, the receptor glutamate ionotropic receptor delta type subunit 2 (GRID2) binds ligand cerebellin 1 precursor (CBLN1) (protein secretion) to transmit the signal to TF POU class 2 homeobox 1 (POU2F1) through SMC2. This upregulates the target gene hematopoietic prostaglandin D synthase (HPGDS) and inhibits the migration and proliferation of CRC cells (Tippin et al., 2012).

In **Figure 5**, lncRNA LOC400043 interacted with MIR133B downregulating its expression. The target genes *SMC2*, *POU2F1*, and *GDNF* are downregulated by MIR133B modified by DNA methylation. Owing to the epigenetic effect, the target gene *SMC2* promotes tumor apoptosis and inhibits the cell cycle; the target gene *POU2F1* inhibits cell proliferation; and the target gene *GDNF* inhibits cell migration and proliferation (Segil et al., 1991; Choudhary et al., 2009; Davalos et al., 2012; Evangelisti et al., 2012; Huang et al., 2014; Guo et al., 2015; Wang et al., 2016b).

### Core Pathways in Mid-Stage to Late-Stage CRC Cells

The core differential pathways between mid-stage and late-stage CRC are shown in **Figure 6**. Firstly, in mid-stage CRC cells, the ligand ATRA (apoptosis signal) binds to the receptor RORB and triggers the signaling to EFEMP1, which is modified by phosphorylation and mutation. Subsequently, the signal is transmitted to the protein KCNK2 and finally reaches the TF MAFG, which is modified by mutation and acetylation. Subsequently, the target gene *MINK1* is upregulated, potentially leading to the migration of cancer cells to mid-stage CRC (Hu et al., 2004). Secondly, the receptor OR2H1 receives the GNAL (Chemical stimulation) signal and transmits it through sequential proteins STK17B, GDNF, and CARM1 to TF ZEB1. In this pathway, STK17B is translocated by phosphorylation and downregulated by *MIR302B*; GDNF is modified by mutation; CARM1 is modified by phosphorylation and methylation; and TF ZEB1 is modified by mutation. Finally, the upregulated *LRRN1* leads to cell proliferation (Hossain et al., 2008).

All pathways are initiated from the common receptor GRID2 between mid-stage and late-stage CRC cells. In the mid-stage CRC cells, the ligand CBLN1 (protein secretion) binds to receptor GRID2 to transmit the signal to TF POU2F1 through SMC2, which is modified by mutation. In turn, TF POU2F1 upregulates target gene *HPGDS* to inhibit cell migration and proliferation (Tippin et al., 2012). After regulated by lncRNA LOC400043, MIR133B modified *SMC2* resulting in the inhibition of cell proliferation and migration (Choudhary et al., 2009; Davalos et al., 2012; Guo et al., 2015). In the late-stage CRC cells, receptor GRID2 binds with the ligand CBLN1 (protein secretion) and sequentially transmits the signal to TF WD repeat domain 4 (WDR4) through proteins cell cycle associated protein 1 (CAPRIN1), secreted protein acidic and cysteine rich (SPARC), protein phosphatase 2 regulatory subunit B’’beta (PPP2R3B), ectonucleotide pyrophosphatase/phosphodiesterase 4 (ENPP4), and single-pass membrane protein with aspartate rich tail 1 (SMDT1). Eventually, TF WDR4 upregulates target gene *LIG3* for DNA repair (Chen et al., 1995).

Notably, the late-stage CRC cells involve two additional pathways. In one pathway, receptor glycoprotein V platelet (GP5) binds ligand von Willebrand factor (VWF) (extracellular matrix organization) to activate TF aryl hydrocarbon receptor (AHR) through a cascade of proteins stomatin (STOM), SLC16A, SH3, and cysteine rich domain (STAC), major facilitator superfamily domain containing 2B (MFSD2B), GDNF, arylsulfatase F (ARSF), and ANGEL2. The protein GDNF, which also appears in mid-stage CRC cells pathway is modified by mutation. Finally, TF AHR upregulates target gene *acidic nuclear phosphoprotein 32 family member E* (*ANP32E*) modified by DNA methylation to promote cell proliferation, migration and DNA repair (Obri et al., 2014). In the other pathway, ion *K+* binds to receptor potassium voltage-gated channel subfamily Q member 2 (KCNQ2) and transmits the signaling to TF LIG1 through a cascade of proteins, namely peptidylprolyl isomerase F (PPIF), KCNK2, DNA polymerase theta (POLQ), zinc finger protein 37B (ZNF37BP), SIM1, and HUS1. The second pathway present in both mid-stage and late-stage CRC cells; POLQ is modified by mutation (Lin et al., 2000; Nilsson et al., 2001); target gene *p53-induced death domain protein 1* (*PIDD1*) is downregulated by TF LIG1 which promotes tumor apoptosis.

## Discussion

### Genetic and Epigenetic Progression Mechanisms of Early-Stage to Mid-Stage CRC *via* Cell Apoptosis and Migration

By applying the systems biology approach and PNP method, we constructed the core pathways to investigate the genetic and epigenetic carcinogenic mechanisms of CRC, as shown in **Figure 5**. In the left-pathway in the early-stage CRC cells, the ligand PTK6 binds to the receptor STAP2 phosphorylated in the hypoxic microenvironment (Semenza, 2016). While STAP2 is modified by phosphorylation, the signal is transmitted to cascade proteins TUBB1, armadillo repeat containing 8 (ARMC8), and MAP4K1 (Fujita et al., 2014; Cho et al., 2017). At this point, with signal coupling from SIM1, the pathway activates the MAPK signaling pathway which is related to the control of immune response. MAP4K1 interacts with TF TP53 modified by deubiquitination. Following the modification of TP53 by deubiquitination, it may upregulate *MUC2*, leading to the progression of CRC. The ubiquitinated TF TP53 inhibits apoptosis and downregulates target gene *MUC2* (Malkin et al., 1990; Ghosh et al., 2004). Furthermore, the main cellular functions of *MUC2* are to protect the colon from disease, including the activation of inflammation and immune response and the inhibition of migration due to DNA methylation. Unfortunately, TF TP53 is eventually modified by mutation and deubiquitination that will affect the expression level of *MUC2* to cause tumorigenesis. However, *MUC2* with epigenetic DNA methylation exhibits lower expression to prevent CRC cells from progressing to the mid stage (Moehle et al., 2006; Cobo et al., 2015).

The next pathway begins with the ligand GRB2 in human B lymphocytes, which phosphorylates the receptor SHC3 after binding. This process activates two pathways: (1) through SIM1 to crosstalk with the MAPK pathway *via* protein MAP4K1 (Magrassi et al., 2005; Tashiro et al., 2009; Azzalin et al., 2014), and (2) transmission of the signal to KLHL15, which is modified by ubiquitination and activation of TF TRIM65. This upregulates target gene *CELF2*, which activates apoptosis, to inhibit cell proliferation and migration. KLHL15 effected by epigenetic changes stabilizes the expression level to avoid progression to mid-stage CRC (Ferretti et al., 2016; Wang et al., 2016a). Therefore, we concluded that the second pathway could inhibit cell migration, proliferation, cell apoptosis to prevent the progression of early-stage CRC to mid-stage CRC.

The third pathway initiates with binding of Mg2+ to the receptor PRKD1, which is modified by phosphorylation and mutation. Subsequently, the signal is transmitted through proteins SIM1 and ZNF260 to KRAS. KRAS mediates ZNF260 to stabilize PRKD1 in this pathway. It has been reported that this interaction occurs through epigenetic silencing due to mutation (Serra et al., 2014). After transmission of the signal to TF TCF3 with DNA migration through RHOF, which is modified by methylation and acetylation (Gouw et al., 2005), it upregulates target gene *NFKBIL1* to activate inflammation and immune response and inhibit the development of CRC (Atzei et al., 2010; Mcallister et al., 2014; Taniue et al., 2016).

The next pathway is initiated with receptor WNT4. Several studies have shown that this pathway could inhibit the proliferation and migration of tumors (Seth and Ruiz I Altaba, 2016; Tang et al., 2018). In the WNT-signaling pathway, ligand WIF1 (signal transduction) binds with the receptor WNT4 to transmit the signal to TF ZEB1 through cascade proteins TUBA1B and TMEM39A. When highly expressed in tumor cells, TUBA1B improves their proliferation. To solve this problem, the acetylation of TUBA1B (also affected by epigenetic methylation) may play an important role in balancing the expression and signal transfer to TF ZEB1 through TMEM39A (Lu et al., 2013). TF ZEB1 is duplicated in both early-stage and mid-stage CRC cells. Its major function is to regulate the apoptosis, migration, and proliferation of cancer cells, as well as the downregulation of target gene *HECTD3*. As *HECTD3* exhibits low expression levels, it may lead to apoptosis of cancer cells and promotion of cell cycle (Shu et al., 2017). Hence, the marked reduction in the expression of HECTD3, caused by DNA methylation, may be beneficial to patients. Unfortunately, TF ZEB1 may undergo mutation during the carcinogenic process. Mutated ZEB1 may result in epithelial–mesenchymal transition (Loboda et al., 2011), progressing CRC to the mid-stage. The final pathway in early-stage CRC cells is initiated from receptor RORB. Some studies have shown that RORB acts as a tumor suppressor. A high expression level of RORB may exert an effect on TF TCF3 to activate a downstream pathway in early-stage CRC (Mühlbauer et al., 2013; Wen et al., 2017). In contrast, a low expression level of RORB may activate another downstream pathway in the mid-stage CRC.

After binding of ligand ATRA to receptor RORB (apoptosis signal), two pathways with epigenetic modifications are activated. In the first pathway, in early-stage CRC, the upregulated CASC2 transmits the signal to HINFP, which is modified by ubiquitination to inhibit tumor growth (Baldinu et al., 2007), and transmits the signal to TF TCF3 through SMG8. Subsequently, SMG8 is modified by phosphorylation to maintain its function in inhibiting cell apoptosis. Finally, the signal is transmitted to TF TCF3 to regulate target gene *NFKBIL1*, directing inflammation and immune response. We concluded that the cellular dysfunctions in early-stage CRC should include immune response, inflammation, and cell apoptosis. In the second pathway, in mid-stage CRC, receptor RORB transmits the signal to TF MAFG through EFEMP1 and KCNK2; epigenetic modifications were also found in this pathway. EFEMP1 is modified by phosphorylation, which activates the mitogen-activated protein kinase/extracellular signal-regulated kinase (MAPK/ERK) signaling pathway (Dou et al., 2016) to control the disordered cell proliferation in mid-stage CRC (Setia et al., 2014). Following activation of the MAPK/ERK pathway, the signal is transmitted through KCNK2 to TF MAFG, which plays a crucial role in this pathway by the modification of acetylation to promote cell migration (Fang et al., 2014; Vera et al., 2017). Subsequently, *MINK1* is upregulated by MAFG. Moreover, previous studies (Hu et al., 2004; Nicke et al., 2005) have shown that *MINK1* is an essential target gene in the MAPK/ERK pathway. Specifically, sustained high expression of *MINK1* is associated with the occurrence of cell migration. Furthermore, there are some proteins and one gene affected by mutation in this pathway. For example, EFEMP1 is modified by mutation; this may inactivate the MAPK/ERK pathway to induce cell proliferation. The next protein is MAFG; following the mutation of TF MAFG, acetylation does not downregulate its expression level. The aforementioned process may inhibit cell migration. In general, *MINK1*, which is regulated by the TF MAFG, exhibits high expression in CRC. However, while *MINK1* is mutated, the expression may be downregulated to inhibit cell migration.

The second pathway in the mid-stage CRC cells starts with receptor OR2H1. When OR2H1 binds ligand GNAL, it may stimulate the G-protein signal pathway to promote cell proliferation. The signal is transmitted through STK17B, which is modified by phosphorylation to control the cell cycle and apoptosis, to GDNF. GDNF is an essential protein related to cell migration in the progression of CRC. Numerous studies (Oppenheim et al., 1995; Evangelisti et al., 2012; Zhang et al., 2017; Fielder et al., 2018) have shown that GDNF would control cell migration. However, while GDNF is mutated in the signaling cascade, it may cause the pathway to be inactive. CARM1 (modified by methylation and phosphorylation) transmits the signal to TF ZEB1, which may be upregulated to cause cell proliferation (Guo et al., 2017; Zhang et al., 2018). TF ZEB1 plays an important role in this pathway to connect the early-stage and mid-stage of CRC. In the mid-stage, ZEB1 may upregulate *LRRN1* to inhibit cell proliferation (Hossain et al., 2008).

The final pathway in the mid-stage CRC cells is activated by receptor GRID2 after binding the ligand CBLN1 (protein secretion). The signal is transmitted to TF POU2F1 through SMC2 to upregulate target gene *HPGDS*, inhibiting cell proliferation and migration. There are two studies (Tippin et al., 2012; Tippin et al., 2014) indicating that high expression of *HPGDS* might inhibit tumor proliferation in normal human cells. However, in CRC, *HPGDS* exhibits a five-fold lower expression (Tippin et al., 2012) to promote tumor migration. The aforementioned processes do not involve mutations. Mutations in this pathway (e.g., receptor GRID2) would lead to its inactivation. Moreover, mutation of the protein SMC2, may impair the natural expression and promote tumor growth.

We also investigated the role of miRNA regulation in the molecular carcinogenic mechanism of mid-stage CRC. As shown in **Figure 5**, some proteins (GDNF, SMC2, and POU2F1) may be translocated through miRNA regulation. One study has indicated that the overexpression of MIR133B in CRC cells induces apoptosis and cell cycle arrest at G1 phase (Lv et al., 2015). However, they have not mentioned that the molecular mechanism involved in the regulation of MIR133B. In this study, we hypothesized that the overexpression of MIR133B would lead to loss of its inhibitory function on other genes. However, accompanying by the regulation of lncRNA LOC400043 and DNA methylation, MIR133B might reverse the overexpression to its natural expression, leading to downregulation of *GDNF*, *SMC2*, and *POU2F1* in this pathway. Furthermore, the high expression of *GDNF* may affect tumor migration. As *GDNF* is downregulated by MIR133B, it inhibits tumor migration and proliferation (Evangelisti et al., 2012; Huang et al., 2014). The expression of *SMC2* modified by MIR133B could downregulate the control of tumor proliferation, migration, and apoptosis (Choudhary et al., 2009; Davalos et al., 2012; Je et al., 2014). *POU2F1* may be overexpressed in CRC cells to cause proliferation. Affected by MIR133B, the expression of *POU2F1* would be downregulated to inhibit cell proliferation. Finally, we found that the cellular dysfunctions in mid-stage CRC include migration and proliferation. The key point of progression from early-stage to mid-stage cancer cells is the mutation of TF ZEB1, which cross-talks in two pathways.

### Genetic and Epigenetic Progression Mechanisms From Mid-Stage to Late-Stage CRC *via* Cell Migration and Proliferation

Late-stage CRC involves widespread metastasis (Jorissen et al., 2009). Only a few studies have investigated late-stage CRC. **Figure 6** shows three core pathways in the late-stage CRC cells. The first pathway starts with receptor GRID2, which also appears in mid-stage CRC cells, and transmits the signal to TF WDR4 to upregulate target gene *LIG3*. *LIG3* has been investigated in studies concerning DNA repair (Murray et al., 2011; Hu et al., 2018). DNA damage and repair are double-edged swords in cancer. DNA damage, coupled with error-prone repair, could drive cancer progression by promoting genomic or genetic instability (Doksani and De Lange, 2016; Hu et al., 2018). Based on our data analysis result, we infer that the DNA repair in response to DNA damage provides a possibility to prevent CRC cells from progressing. However, failures in DNA repair would lead to the deterioration of patient’s condition.

The core pathway contains protein GDNF, which is the critical factor in the progression of CRC. This pathway starts with receptor GP5, which binds ligand VWF (extracellular matrix organization), transmitting the signal to TF AHR through GDNF. AHR regulates cell proliferation (Xie and Raufman, 2015) by upregulating target gene *ANP32E*. The upregulated *ANP32E* may cause tumor migration and proliferation. Our results show that *ANP32E* is modified by DNA methylation to downregulate *ANP32E*, which leads to the promotion of tumor migration and proliferation, as well as DNA repair (Obri et al., 2014).

The last pathway in the late stage starts with receptor KCNQ2 binding with the K+ transmits signal to TF LIG1 through cascade protein KCNK2, which also appears in the mid-stage CRC cells. POLQ, which is prevalent in CRC, is regarded as a tumor promoter because its mutation could cause overexpression to promote cell migration (Higgins et al., 2010). When the signal is transmitted to TF LIG1, it may downregulate target gene *PIDD1* to promote cell apoptosis (Lin et al., 2000; Nilsson et al., 2001). In other words, TF LIG1 is a tumor suppressor which regulates *PIDD1* to promote cell apoptosis. Regulated by lncRNA LOC400043, MIR133B is able to downregulate *SMC2* resulting in the inhibition of cell migration. Of note, mutation of the receptor GRID2 denying the binding of ligand CBLN1 is the key for the progression from mid-stage to late-stage CRC.

### Genetic and Epigenetic Carcinogenic Mechanisms in Early-Stage to Late-Stage CRC Cells

**Figure 7:**
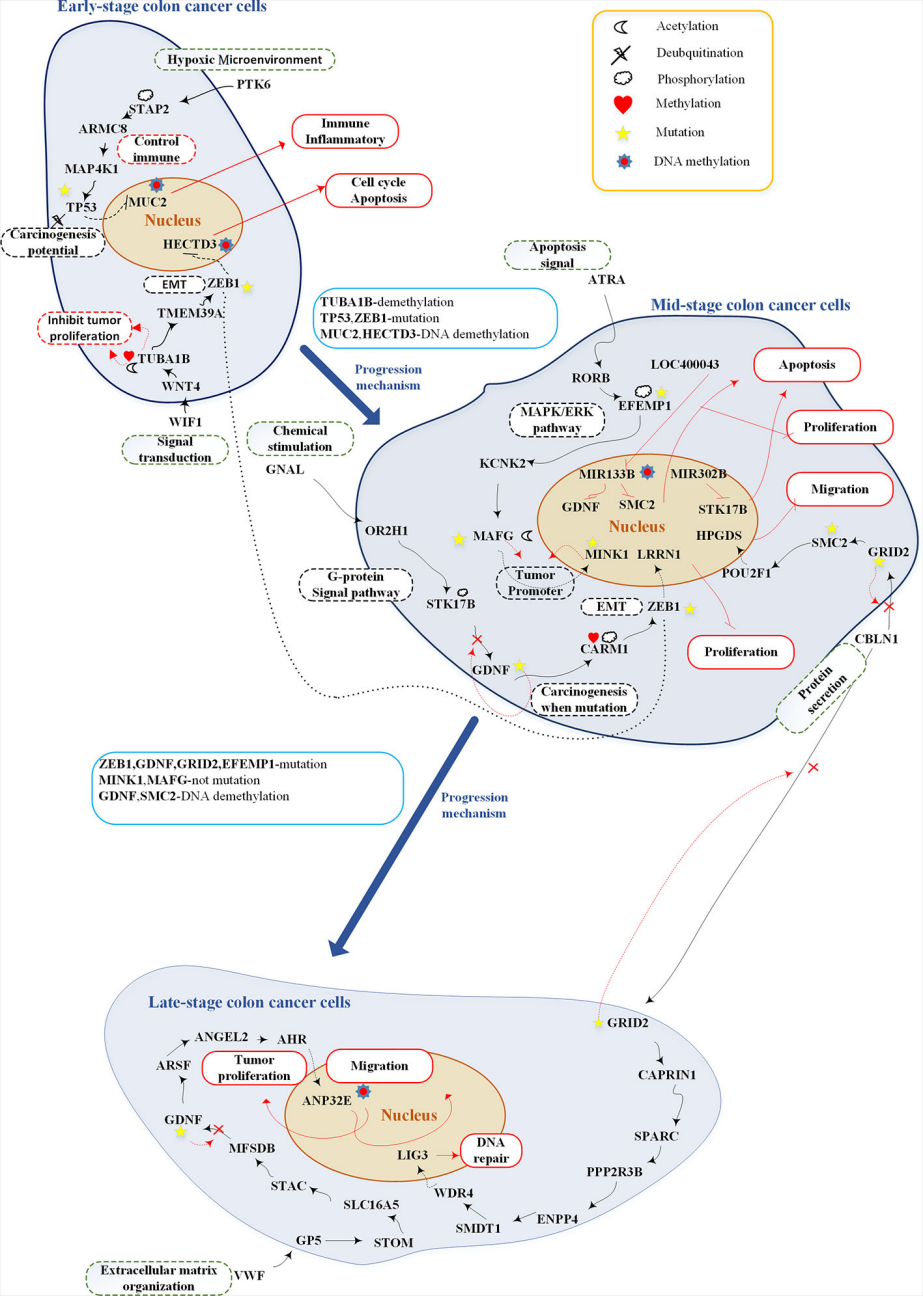
Overview of genetic and epigenetic progression mechanisms from early-stage colon cancer cells to late-stage colorectal cancer cells.The upper part shows the genetic and epigenetic mechanisms of early-stage colon cancer cells; the middle part shows the genetic and epigenetic mechanisms of mid-stage colon cancer cells; the lower part shows the genetic and epigenetic mechanisms of late-stage colon cancer cells. The black lines represent protein-protein interaction; the red lines represent upregulation or downregulation; the black dash lines represent upregulation or downregulation; black dot line represents crosstalk; the red dash lines represent genetic, epigenetic, and mutation effects; red rectangular represents cellular functions; black dash rectangles include pathway description; green dash rectangles represent microenvironment; the red cross indicates mutation which may inactivate the pathway; and the blue rectangles show progression mechanisms.

After analyzing the pathway of each stage of CRC, we recognized some essential pathways and graphically illustrated them in **Figure 7**. Based on the investigation of the core genetic and epigenetic network, we identified some pathogenic biomarkers for the design of multiple drugs against CRC. In early-stage disease, PTK6 (hypoxic environment) binds with STAP2 to activate essential protein MAP4K1 and consequently the MAPK pathway (Fujita et al., 2014; Cho et al., 2017). WIF1 (Signal transduction) binds with WNT4 to activate the WNT signaling pathway (Seth and Ruiz I Altaba, 2016; Tang et al., 2018). *MUC2* and *HECTD3*, modified by DNA methylation, could promote immune response, inflammation, cell cycle, and apoptosis. At this stage, TF TP53 and ZEB1 are two potential factors of tumor progression, which are modified by mutation to cause cell migration (Malkin et al., 1990; Ghosh et al., 2004; Loboda et al., 2011). The mutated TF TP53 and ZEB1 promote the progression of CRC toward the mid stage.

Mid-stage CRC involves three pathways: (1) ligand GNAL (chemical stimulation) binds with receptor OR2H1 to activate the G-protein signaling pathway; (2) ligand ATRA (apoptosis signal) binds with receptor RORB to activate the MAPK/ERK pathway (Setia et al., 2014); and (3) ligand CBLN1 (protein secretion) binds with receptor GRID2 to activate the CREB pathway (Hui et al., 2014). The most important carcinogenic mechanism and cellular dysfunctions at this stage of CRC are as follows: MIR133B is modified by DNA methylation to downregulate GDNF and SMC2, inhibiting tumor proliferation and migration. Target gene MINK1 and its TF MAFG are two tumor promoters (Hu et al., 2004; Nicke et al., 2005; Fang et al., 2014; Vera et al., 2017). Affected by mutations, their expression may be reduced to prevent tumor migration. In other words, they would abolish their tumor promoting activity. GDNF, EFEMP1, and GRID2 play important roles in carcinogenesis at this stage. Following the occurrence of mutations within the cascade signaling pathway, these pathways may be inactivated and accelerate tumor migration.

The late-stage CRC cells involve two core pathways: (1) ligand CBLN1 (protein secretion) binds with receptor GRID2; and (2) ligand VWF (extracellular matrix organization) binds with receptor GP5. In the first pathway, the target gene *LIG3* controls DNA repair. In the second pathway, the target gene *ANP32E* is modified by DNA methylation to downregulate its expression and retard tumor migration (Li et al., 2017; Xiong et al., 2018).

### Design of Multiple-Molecule Drugs for Preventing the Progression From Early-Stage to Mid-Stage and Mid-Stage to Late-Stage CRC by Querying Connectivity Map

According to the analyzed results of core signaling pathways for three stages of CRC, genetic, and epigenetic biomarkers are identified as drug targets for designing multiple-molecule drug to prevent progression from early-stage to mid-stage and mid-stage to late-stage CRC. Connectivity Map (CMap) build 02, a project developed by Broad Institute, contains 6100 instances with 1,309 drugs and 156 concentrations on five cell lines (Musa et al., 2017). By querying CMap build 02, we suggested three potential compounds with high negative connectivity scores and combined them with the known drugs of CRC for preventing progression from early stage to mid stage of CRC and mid stage to late stage of CRC, respectively. The correlation coefficients between the concentrations of drugs and the gene expression levels indicate the relationship between drugs and genes. If the correlation coefficient is >0, the gene is upregulated by treatment with the drug; if the correlation coefficient is <0, the gene is downregulated by treatment with the drug.

We selected one genetic biomarker and four epigenetic biomarkers in **Table 2**: the genetic biomarker is MINK1 and the epigenetic biomarkers are MUC2, HECTD3, GDNF, and SMC2. The epigenetic biomarkers were selected owing to their high expression level, as a consequence of alterations in epigenetic regulation resulting in cell migration and proliferation. Moreover, MINK1 exhibits a high expression level, resulting in cell migration in CRC. We selected two known drugs used in the treatment of CRC, (i.e., 5-fluorouracil and oxaliplatin), and three potential compounds (i.e., mesalazine, dexverapamil, and sulindac) by querying CMap to restore the normal expression of five target genes (**Table 2**). The results showed that the expression of five target genes were decreased through the treatment with the proposed potential compounds. Among them, it is noted that there are several evidences demonstrating the ability of mesalazine and its derivative to interfere with intracellular signals involved in CRC cell growth (Bus et al., 1999; Lyakhovich and Gasche, 2010; Stolfi et al., 2012). Meanwhile, ClinicalTroals.gov identifier NCT02077777, has shown mesalazine completed phase II clinical trial based on definitions developed by the U.S. Food and Drug Administration (FDA) for chemopreventive action of mesalazine on CRC. The dexverapamil has been regarded as chemosensitizer, which is a small molecule making tumor cells be sensitive to the chemotherapeutic agents (Weinlander et al., 1997; Hu et al., 2016). Moreover, there is one study showing sulindac could inhibit CRC cell growth and downregulate specificity protein transcription factors (Li et al., 2015). Based on these results, we suggest that combining three potential drugs with two known CRC drugs may alleviate the rate of deterioration from early-stage to mid-stage CRC.

**Table 2:** Drug targets and the corresponding multiple-molecule drug for the therapeutic treatment from early-stage to mid-stage colorectal cancer.

Expression (before treatment)	Targets	5-fluorouracil, oxaliplatin (known)	mesalazine	dexverapamil	sulindac	Expression (after treatment)
+	MUC2		−	−	−	−
+	GDNF		−	−	−	−
+	HECTD3		−	−	−	−
+	MINK1		−	−	−	−
+	SMC2		−	−	−	−

+ denotes activation and − denotes repression.

As shown in **Table 3**, we identified four genetic biomarkers and one epigenetic biomarker. The genetic biomarkers are LIG3, STK17B, MINK1, and LRRN1, and the epigenetic biomarker is SMC2. According to the above analysis, MINK1, STK17B, LRRN1, and SMC2 exhibit high expression level to promote cell migration in CRC; LIG3 has a high expression level to cause failure in DNA repair. We selected two known drugs of CRC (i.e., 5-fluorouracil and oxaliplatin), and proposed three potential compounds (i.e. valproic acid, estradiol, and gefitinib) by querying CMap to restore the normal expression of five targets. Notably, the valproic acid not only inhibits CRC cells growth through cell cycle modification but also has the ability to reverse aberrant DNA methylation partially (Strey et al., 2011; Brodie and Brandes, 2014; Bressy et al., 2017). Moreover, estradiol has been found that it reduced proliferation and apoptosis in CRC (Sasso et al., 2019). For gefitinib, there are a number of phase I and II studies investigating the effects caused by the combination with standard 5-fluorouracil (5-FU)-based regimes with response rates ranging from 25 to 59%, although these trials did not include chemotherapy-resistant individuals (Kuo et al., 2005; Cho et al., 2006; Hofheinz et al., 2006; Wolpin et al., 2006; Stebbing et al., 2008). Moreover, ClnicalTrials.gov identifier NCT00025350, has completed phase II trial for using gefitinib in patients with recurrent metastatic CRC. According to these results, we suggest that the combination of three potential compounds with two known drugs used against CRC as a multiple-molecule drug may retard the rate of deterioration from mid-stage to late-stage CRC.

**Table 3:** Drug targets and the corresponding multiple-molecule drug for the therapeutic treatment of mid-stage and late-stage colorectal cancer.

Expression (before treatment)	Targets	5-fluorouracil, oxaliplatin (known)	valproic acid	estradiol	gefitinib	Expression (after treatment)
+	SMC2		−	−	−	−
+	MINK1		−	−	−	−
+	LIG3		−	−	−	−
+	STK17B		−	−	−	−
+	LRRN1		−	−	−	−

+ denotes activation and − denotes repression.

### The Model Evaluations and Limitations of Systems Biology Approaches to Infer the Core Signaling Pathways of CRC

In drug discovery, biomarker identification is an important problem. Ligand binds to receptor, which trigger downstream signaling cascade, and results in the progression of tumor cells. In this study, in order to investigate the core signaling pathways of CRC for identifying essential biomarkers, we leveraged microarray CRC dataset to construct real GWGENs by the reversed engineering method. Afterwards, we applied Akaike’s information criterion (AIC), which could help us to prune the false positives of regulations and interactions in the GWGENs and conquer the overfitting and under fitting problems. To evaluate our proposed models, we found another independent dataset, Colorectal Adenocarcinoma (TCGA, PanCancer Atlas) (Hoadley et al., 2018), and calculated the AIC values for the common symbols. Subsequently, we executed random permutation for 1,000 times on our original dataset (GSE14333). Here, we would like to know what percentage of common symbols in the independent dataset could have significant p-value. In other words, taking one common symbol for example, if the AIC value of common symbol could be lower than all of the AIC values after random permutation in the original dataset, we would say that the common symbol has significant p-value (p-value < 0.001). According to our model evaluation results for each stage of CRC in the independent dataset, there are 0.5847, 0.5170, and 0.5867 percentage of common symbols with significant p-value, respectively. The corresponding model evaluation analysis code could be found in the Github link (https://github.com/lab619nthu/Validation.git). Moreover, we also found that the symbol with numerous edges was prone to have the higher p-value than the original dataset. This phenomenon implies that the experimental condition change would have severe effects on symbol owning lots of edges.

It is noted that not all of the pathway analysis of proteins (**Tables 4**–**6**) have been proved to be associated with CRC (for example, the HCM pathway shown in **Supplementary Table 2**). We conclude multiple reasons resulting in such finding. First, it is known that microarray dataset is very noisy. Considering the fact that most of the models are linear in our pipeline, for some context, the true signal may be buried in the accumulated noise due to the high dimensionality of the dataset. In future, we would like to enhance our pipeline and try to minimize such effect as much as possible. Secondly, cancer cells could utilize cellular programs which are different from normal cells to survive in the stressful microenvironment. For example, reactivation of cancer-testis antigen *BAP31* has been identified to promote proliferation and invasion in cervical cancer (Dang et al., 2018). Therefore, we could not totally exclude the possibility that the HCM pathway promotes the progress of disease as cancer cells could utilize unusual cellular programs to survive. The ectopic expressions may have something to do with the rewiring of cancer cellular program. Currently, we only put focus on gene expression. In future, we would like to integrate multiple types of molecular data into our pipeline and have more exploration on this.

**Table 4:** Pathway analysis of proteins in the real genome-wide genetic and epigenetic network (GWGEN) of early-stage colon cancer.

KEGG pathway	Numbers	p-value
Metabolic pathways	714	3.3*10^-14^
Neuroactive ligand-receptor interaction	186	2.0*10^-10^
Calcium signaling pathway	109	7.2*10^-4^
Cytokine-cytokine receptor interaction	132	4.7*10^-3^
Hypertrophic cardiomyopathy (HCM)	47	3.8*10^-2^
Pyruvate metabolism	26	5.0*10^-2^

In the real GWGEN of early-stage colon cancer, the top six enriched Kyoto Encyclopedia of Genes and Genomes (KEGG) pathways were: metabolic pathways, neuroactive ligand-receptor interaction, calcium signaling pathway, cytokine-cytokine receptor interaction, hypertrophic cardiomyopathy (HCM), and pyruvate metabolism.

**Table 5:** Pathway analysis of proteins in the real genome-wide genetic and epigenetic network (GWGEN) of mid-stage colon cancer.

KEGG pathway	Numbers	p-value
Cell adhesion molecule (CAM)	7	6.2*10^-3^
African trypanosomiasis	3	5.7*10^-2^
Extracellular matrix (ECM)-receptor interaction	4	8.2*10^-2^

In the real GWGEN of mid-stage colon cancer, the top three enriched Kyoto Encyclopedia of Genes and Genomes (KEGG) pathways were: cell adhesion molecules (CAMs), African trypanosomiasis, and PECM-receptor interaction.

**Table 6:** Pathway analysis of proteins in the real genome-wide genetic and epigenetic network (GWGEN) of late-stage colon cancer.

KEGG pathway	Numbers	p-value
MAPK signaling pathway	7	3.4*10^-2^
Calcium signaling pathway	6	2.8*10^-2^
HIF-1 signaling pathway	4	6.7*10^-2^
Signaling pathways regulating pluripotency of stem cells	5	4.4*10^-2^
Amyotrophic lateral sclerosis (ALS)	3	8.2*10^-2^
Pathways in cancer	10	1.2*10^-2^

In the real GWGEN of late-stage colon cancer, the top six enriched Kyoto Encyclopedia of Genes and Genomes (KEGG) pathways were: mitogen-activated protein kinase (MAPK) signaling pathway, calcium signaling pathway, HIF-1 signaling pathway, signaling pathways regulating pluripotency of stem cells, amyotrophic lateral sclerosis (ALS), and pathways in cancer.

## Data Availability Statement

The genome-wide microarray raw data were downloaded from the Gene Expression Omnibus database (https://www.ncbi.nlm.nih.gov/geo/query/acc.cgi), including 290 primary colorectal tumor samples (GSE14333).

## Author Contributions

Conceptualization: S-JY and S-WC. Methodology: S-JY, S-WC, and B-SC. Software: S-JY and S-WC. Validation: S-JY. Formal Analysis: S-JY and S-WC. Investigation: S-WC. Data Curation: S-WC. Writing—Original Draft Preparation: S-JY. Writing-Review and Editing: S-JY, S-WC, and B-SC. Visualization: S-JY and S-WC. Supervision: B-SC. Funding Acquisition: B-SC.

## Funding

This research was funded by the Ministry of Science and Technology (grant number: MOST 107-2221-E-007-112-MY3).

## Conflict of Interest

The authors declare that the research was conducted in the absence of any commercial or financial relationships that could be construed as a potential conflict of interest.
