# Supplementary material for: Investigation of the Genome-Wide Genetic and Epigenetic Networks for Drug Discovery Based on Systems Biology Approaches in Colorectal Cancer
Source: Front Genet. 2020 Mar 6;11:117. doi: 10.3389/fgene.2020.00117 (PMC7068214; doi:10.3389/fgene.2020.00117)
Supplement: Supplementary file 2 [file Presentation_1.pdf]

Supplementary Materials

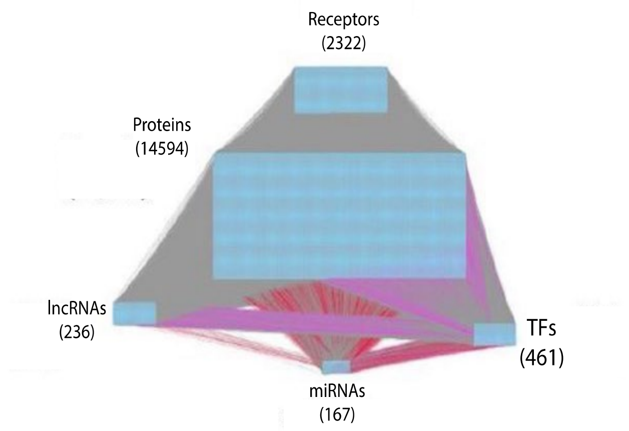

Figure S1. Real GWGEN of early-stage progression of colon cancer. The real GWGEN of early-stage colon cancer is pruned from candidate GWGEN by system identification and system order detection method through the corresponding microarray dataset. Among the real GWGEN of early-stage CRC, there are 2322 receptors, 14594 proteins, 236 lncRNAs, 167 miRNAs, and 461 TFs. The gray lines represent edges in PPIN; the purple lines represent edges in GRN; the red lines represent miRNA regulations.

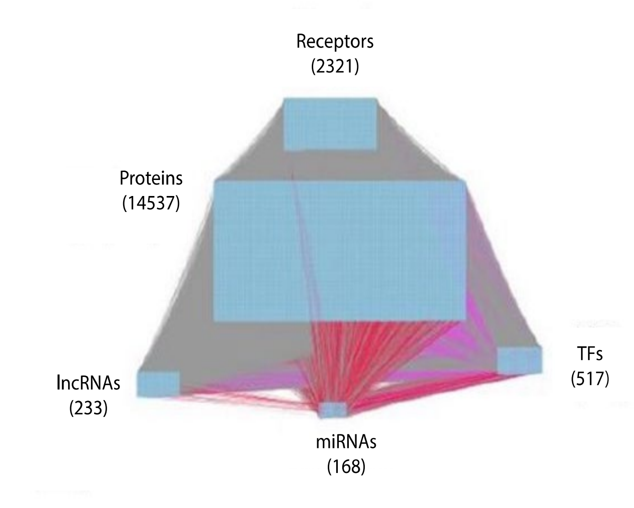

Figure S2. Real GWGEN of mid-stage progression of colon cancer. The real GWGEN of mid-

stage colon cancer is pruned from candidate GWGEN by system identification and system order detection method through the corresponding microarray dataset. Among the real GWGEN of mid-stage CRC, there are 2321 receptors, 14537 proteins, 233 lncRNAs, 168 miRNAs, and 517 TFs. The gray lines represent edges in PPIN; the purple lines represent edges in GRN; the red lines represent miRNA regulations.

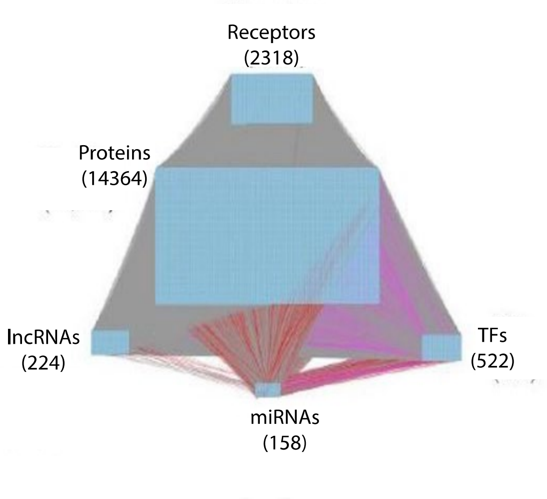

Figure S3. Real GWGEN of late-stage progression of colon cancer. The real GWGEN of late-stage colon cancer is pruned from candidate GWGEN by system identification and system order detection method. Among the real GWGEN of late-stage CRC, there are 2318 receptors, 14364 proteins, 224 lncRNAs, 158 miRNAs, and 522 TFs. The gray lines represent edges in PPIN; the purple lines represent edges in GRN; the red lines represent miRNA regulations.
